# The benefits of mystery in nature on attention: assessing the impacts of presentation duration

**DOI:** 10.3389/fpsyg.2014.01360

**Published:** 2014-11-25

**Authors:** Andrew M. Szolosi, Jason M. Watson, Edward J. Ruddell

**Affiliations:** ^1^Department of Recreation and Sport Pedagogy, Ohio UniversityAthens, OH, USA; ^2^Department of Psychology, University of UtahSalt Lake City, UH, USA; ^3^Department of Parks, Recreation, and Tourism, University of UtahSalt Lake City, UH, USA

**Keywords:** mystery, fascination, attention restoration theory, recognition memory, mediation testing

## Abstract

Although research has provided prodigious evidence in support of the cognitive benefits that natural settings have over urban settings, all nature is not equal. Within nature, natural settings that contain mystery are often among the most preferred nature scenes. With the prospect of acquiring new information, scenes of this type could more effectively elicit a person's sense of fascination, enabling that person to rest the more effortful forms of attention. The present study examined the direct cognitive benefits that mystery in nature has on attention. Settings of this sort presumably evoke a form of attention that is undemanding or effortless. In order to investigate that notion, participants (*n* = 144) completed a Recognition Memory Task (RMT) that evaluated recognition performance based on the presence of mystery and presentation duration (300 ms, 1 s, 5 s, and 10 s). Results revealed that with additional viewing time, images perceived high in mystery achieved greater improvements in recognition performance when compared to those images perceived low in mystery. Tests for mediation showed that the effect mystery had on recognition performance occurred through perceptions of fascination. Implications of these and other findings are discussed in the context of Attention Restoration Theory.

## Introduction

One of the many benefits often ascribed to interactions with nature is their ability to provide a person with rest and relief from the demands of everyday life (Kaplan and Kaplan, 1982; Knopf, 1987). A long walk in a nearby park, a view of a snow-capped mountain, or even the simple act of tending to the garden can be enough to alleviate the mental fatigue that may escalate throughout the course of a day. Research in this area has amassed a considerable amount of evidence in support of the beneficial effects that nature can have on a person's cognitive functioning (Hartig et al., 2003; Berto, 2005; Berman et al., 2008).

All nature however, is not equal. Certain types of natural settings are likely more supportive or effective at providing a person with the kind of rest needed to facilitate a state of mental recovery. Natural settings that contain patterns of mystery may be particularly effective at this aim, as previous research has shown mystery to be a strong predictor for environmental preference (Herzog and Kropscott, 2004; Stamps, 2004; Herzog, 2007). Consider a partially obstructed view, a view common to many nature or park settings. Scenes of this sort give the impression that there is more to gain by going deeper into the setting. The possibility of acquiring new information captures a person's interest, prompting that person to look more carefully through the leafy branches and slim tree trunks of a forest. The fact that the view is vaguely seen makes it all the more elusive, distant, and fascinating. Such sources of fascination presumably serve as a basis for resting attention (Cimprich, 1992; Kjellgren and Buhrkall, 2010).

Efforts to evaluate the effects that nature has on resting and restoring attention have often relied on approaches in which setting exposure occurs between a mental load task and a specific assessment of attention (Tennessen and Cimprich, 1995; Berto et al., 2010). Although approaches of this type can indicate the extent to which a particular setting can facilitate recovery, they do not necessarily implicate the cognitive processes that contribute to that recovery. In the present study, we adopted an alternative method in which setting exposure was part of the attention-related task. The intent of this approach and the current study was not to address restoration *per se*, but to examine the underlying mechanisms that presumably contribute to that outcome. The prevailing assumption is that settings that are cognitively restorative tend to engage forms of attention that are more effortless, allowing the more effortful mechanisms of attention an opportunity to rest (Kaplan, 1995). Attention Restoration Theory (ART) offers an explanation for why certain settings may facilitate the activation of these less demanding forms of attention (Kaplan, 1995).

ART, developed by the Kaplans (Kaplan and Kaplan, 1989), has served as the theoretical framework to help guide and explain why interactions with certain settings may lead to a restorative experience. As a concept, the restorative experience draws on the notion that after periods of prolonged use or under conditions of cognitive load, a person's capacity to direct attention can become fatigued (Hartig and Staats, 2006). Researchers have often referred to this condition as directed attention fatigue (Kaplan, 2001; Felsten, 2009). Experiences of directed attention fatigue can be significant, interfering with a person's ability to function effectively in everyday life. As a cognitive capacity, directed attention allows a person to block out competing distractions in order to sustain focus during purposeful activity (Posner and Snyder, 1975; Kaplan and Berman, 2010). When the demands on this capacity become too overwhelming, people often seek out settings that offer some sense of respite or relief. For many people, interacting with nature fulfills that role.

According to ART, recovery from directed attention fatigue will occur to the extent that four factors are present in the person-environment interaction (Kaplan, 1995; Staats et al., 2003). To that end, for an experience to be restorative a person must garner a sense of physical and cognitive distance from the distractions and routines that place demand on his or her capacities (*being away*). Although gaining some degree of distance is important, restoration critically depends upon the presence of stimuli that are inherently interesting (*fascination*); a point examined further in the section that follows. Stimuli perceived as fascinating seemingly call forth a form of attention that is less demanding or effortless. By avoiding circumstances that require mental effort, a person can thereby rest the more effortful forms of attention (Berman et al., 2008; Kaplan, 1992, 1995). For that rest to be something other than fleeting though, a setting must also comprise a sense of scope and connectedness (*extent*). That is, the person-environment interaction must be rich and coherent enough to not only capture attention, but also sustain it.

The final restorative component addresses the need for a person's inclinations and purposes to be congruent with the requirements or demands imposed by a setting (*compatibility*). The degree to which a setting is compatible has a direct effect on human functioning (Herzog et al., 2011). Settings that do not support a person's intentions tend to require a considerable amount of mental effort. Although the restorative factors discussed here can exist in all types of settings, natural settings tend to hold all four at high levels (Herzog et al., 1997; Hartig et al., 2003). This is especially true for fascination, as nature is full of stimuli that tend to intrinsically capture a person's interest.

The term, fascination, draws on a distinction first proposed by James (1890). That distinction revolved around two mechanisms of attention: voluntary and involuntary. Voluntary attention is the willful act of directing mental effort so as to meet certain prescribed goals. In some situations, we may find that the stimulus patterns in an environment are essential to achieving a specific purpose, but yet fail to hold our interest. Voluntary or top-down processing allows a person to employ mental effort in order to ignore irrelevant information and focus solely on those items that are most salient. In contrast, involuntary attention (*fascination*) is largely a function of the interest or attraction that is present within an environment (Posner et al., 1980). On these occasions, the environmental patterns are so appealing that the activation or selection of attention occurs almost effortlessly. As a result, the processing of information is more bottom-up oriented.

According to Kaplan and Talbot (1983), human fascination tends to derive from certain cognitive contents and processes. The contents that frequently elicit fascination are often those objects that a person perceives as great value or great danger, or that hold evolutionary significance such as water, fire, or greenery (Kaplan, 1978). The processes that tend to engage attention more effortlessly are those that facilitate understanding or involvement (Kaplan and Kaplan, 1982). As people interact with a setting that offers both fascinating contents and processes, there are fewer demands placed on a person's processing capacity (Berto et al., 2008).

With the suggestion that there is more to see, natural settings that contain mystery can be very compelling. According to Kaplan and Kaplan (1982), the source of that fascination derives from strong biases early humans formed for visual information. As a species that did not rely on physical prowess for survival, humans tended to prefer environments that could facilitate understanding and involvement (Kaplan, 1987). Too much familiarity, and a person can become bored or tired. Too engaging, and a person could very easily experience feelings of anxiety or frustration. Settings that allow for a person to make sense while also promoting opportunities for exploration can not only engage, but also sustain a person's interest or fascination.

Mystery refers to those settings where a portion of the visual landscape is obstructed, enticing a person to go further (Hammitt, 1980; Kaplan and Kaplan, 1982). A bend in the trail, a view partially concealed by foliage, or a stream that meanders out of sight all possess attributes related to mystery (Gimblett et al., 1985). Scenes of this type often provide the prospect to acquire additional information. This in turn can engage a person's interest and enhance one's sense of involvement. Although there is ample evidence demonstrating the benefits nature has over urban environments (Tennessen and Cimprich, 1995; Herzog et al., 1997; Hartig and Staats, 2006), few studies have teased out the impact that specific scenic qualities within nature have on attention.

Previous studies have documented well the benefits that interactions with nature can have on a person's cognitive functioning (Kuo and Sullivan, 2001; Hartig et al., 2003; Berto, 2005; Berman et al., 2008; Taylor and Kuo, 2009). In many of those studies, the experimental design revolved around examining changes in performance for measures intended to evaluate attentional functioning. In the present research, we employed the use of a recognition memory task (RMT) in order to assess the mental workload that images perceived high or low in mystery had on a person's cognitive capacity. One of the major assumptions underlying ART is that recovery from directed attention fatigue is contingent upon resting that capacity (Kaplan, 1995).

In our use of the RMT, presentation duration served as an independent variable. In varying the amount of time a group of subjects had to study presented images, we not only were able to simulate demand on attention, but also address the extent to which certain images might evoke more automatic forms of processing. Faster presentation durations often correlate with mechanisms of attention that are more automatic in quality. With duration held as a constant for a person carrying out the RMT, performance on the RMT should vary as a function of the cognitive costs associated with a particular processing component (Barrouillet et al., 2004). For this study, the processing components were the images that appeared on the computer screen. Evaluations of task performance overall, as well as examining performance for both studied (hits) and non-studied (false alarm) images offered insight into the underlying mechanisms of attention activated during the RMT. That is, to what extent do scenes containing patterns of mystery engage or activate a type of attention that is more effortless in form? Further affirmation of these potential benefits occurred through our examination of the remember-know judgments made following recognition decisions.

In sum, we experimentally tested a series of hypotheses that aimed to understand the effects that high and low mystery nature images had on a person's attention. Initially, we expected to see differences in participants' recognition performance as a function of presentation duration and scene type (Hypothesis 1). We then anticipated that scene type would significantly predict recognition performance in that images perceived high in mystery would lead to greater rates of recognition performance (Hypothesis 2). In an attempt to explain this outcome, we first tested the supposition that scene type would predict levels of perceived fascination; images perceived high in mystery would result in higher levels of perceived fascination (Hypothesis 3). Finally, we expected that the effects of scene type on recognition performance would occur through perceptions of fascination (Hypothesis 4).

## Materials and methods

### Participants

A total of 229 introductory psychology students (51% female) received partial course credit for their participation in the study. Participant ages ranged from 18 to 54 (*M* = 22, *S.D*. = 5.19) with the greatest percentage of participants being in their freshman year of college (35.8%). Prior to participating in the study, all students were required to provide informed consent in accordance with the university's institutional review board.

### Research design

Students involved in the study participated in one of three experimental phases. Initial efforts focused on establishing a set of images that best represented the extremes of trail scenes containing attributes commonly related to mystery (Gimblett et al., 1985). After obtaining such images, those images were then incorporated into the RMT. The third and final phase of the study involved an additional sample of participants assessing the same images integrated into the RMT for perceived fascination. Taken collectively, data obtained from each study phase provided a means by which to assess whether scores for fascination served as the generative mechanism through which mystery influenced the rate of recognition memory performance for images presented as part of the RMT.

### Study phases

#### Mystery rating (phase 1)

Phase 1 of the study involved 38 students norming a set of nature trail scenes for the presence of mystery. Specific focus was devoted to obtaining images that reflected patterns perceived high in mystery and patterns perceived low in mystery (i.e., screening, distance of view, etc.). To accomplish that aim, participants viewed 160 images displayed on a computer screen one at a time. For each image presented, participants provided a response rating denoting the extent to which a setting promised more to be seen if they could have walked deeper into that setting (0 = not at all, 6 = Very much so). The evaluation of the presented images was self-paced. The following scenic assessment for mystery demonstrated a high level of internal consistency with a Cronbach's alpha of 0.92.

#### Recognition memory task (phase 2)

Using a 2 × 4 experimental design, the RMT examined the effect that scene type (high vs. low mystery) and presentation duration (300 ms, 1 s, 5 s, and 10 s) had on participants' (*n* = 144) recognition memory performance. Presentation duration served as a between subjects variable, in which there were 36 participants in each duration. Scene type was manipulated within-subjects. Participants carried out the RMT in two parts: a study portion and a test portion. As an intentional learning task, participants were asked to study and memorize each image to the best of their ability. During the study portion of the task, participants viewed one of two subsets of 40 images, in which a computer screen randomly displayed each image for a specific duration. For a given duration, half of the participants viewed images from subset A at study, while the other half viewed images from subset B at study. This counter-balancing of images helped to ensure that participants' performance on the RMT was not merely an artifact of presenting certain images during the study portion of the task. Each subset comprised images from the two scene types examined; 20 images perceived to be low in mystery and 20 images perceived to be high in mystery. After the presentation of an image, a 100 pixel high by 100-pixel wide fixation point appeared for 200 ms.

During the test portion of the task, participants viewed the same 40 images randomly intermixed with the 40 new images (images from either Subset A or Subset B). Similar to the study portion of the RMT, the 40 new images included 20 images perceived to be low in mystery, and 20 images perceived to be high in mystery. For each image presented, participants had to decide whether or not an image was one they had previously seen (OLD) or an image that they were seeing for the first time (NEW). Participant responses yielded an accuracy score for each test image. OLD images correctly identified as OLD were grouped as hits. NEW images incorrectly identified as OLD were categorized as false alarms. In order to obtain a measure of recognition memory performance, participants' hit and false alarm rates for each scene type were calculated. Subtracting a participant's hit rate from his or her false alarm rate yielded a corrected recognition rate for each scene type. The rate of corrected recognition served as the dependent variable as it reflected a more accurate estimation of recognition performance.

In addition to assessing recognition memory performance, the RMT also provided a means by which to evaluate the strength of particular memory trace by asking participants to make remember-know judgments (Watson et al., 2003). If a participant responded that an image was “OLD” (meaning that it was presented during the study portion of the experiment), participants were then prompted to make a remember/know judgment for that image. A “REMEMBER” response indicated that there was something specific a participant remembered about the test image. A “KNOW” response indicated that a participant could not recollect any contextual details for a test image, but possessed a sense of familiarity that allowed that participant to be reasonably confident that the test image was presented during the study session.

#### Perceived fascination (phase 3)

A total of 47 students participated in Phase 3 of the study. Similar to norming a set of images for mystery, this phase focused on obtaining response ratings of perceived fascination for each of the 80 test images that appeared in the RMT. The study used a modified or shortened version of the fascination subscale originally designed as part of the Perceived Restorativeness Scale (Hartig et al., 1996). The scale used for this study consisted of three items that comprised: “This place has qualities that fascinate me,” “I would like to spend more time looking at the surroundings here,” and “My attention is drawn to many interesting things here.” Items selected for inclusion as part of this study phase drew on factor analysis results presented in Hartig et al.'s (1996) earlier work. For each image presented on the computer screen, participants indicated to what extent the statements described the experience he or she was having while viewing a presented image (0 = Not at all, 6 = Very much so). Again, the evaluation of the presented images was self-paced. With a Cronbach's alpha of.96, the modified fascination subscale demonstrated a high level of internal consistency.

#### Measures of individual differences

In order to assess the success of randomly assigning participants to study phases, we included certain measures of individual difference as part of the study. Those measures included the Automated Operation Span Task (Aospan) and a slightly modified Dissociative Experience Scale (DES). A brief description of each measure follows.

Designed to assess working memory capacity, the Aospan is a computer based, mouse driven task that requires participants to solve a series of basic math operations that necessitate a “true” or “false” response. While completing this task, participants are also tasked with trying to remember the order of a set of unrelated letters. For the purpose of this study, we used participants' Aospan total score as an indicator of the attentional resources one had available to them when carrying out a cognitively challenging task (Unsworth et al., 2005). That score comprised the total number of correct letters in the correct position. With a.83 test-retest reliability coefficient, the Aospan is considered to be a highly reliable instrument.

The inclusion of the DES provided some additional insight into whether or not certain participants might have been predisposed toward involuntary shifts in attention (Bernstein and Putnam, 1986). As a self-report measure, the DES consists of 28 items that address feelings of derealization, depersonalization, as well as absorption. In the original measure, participants indicated their response to each item by placing a slash along a line numerically anchored. For the present study, participants denoted their response for each item in accordance to the provided semantic scale (Never, Rarely, Average, Frequently, Always). Each of the semantic ratings received a numerical value from 1 to 5. Using these values, we then calculated a mean DES score based on the sum average of participants responses to each of the items in the DES. In this study, the DES demonstrated strong reliability with a Cronbach's alpha of 0.89.

### Procedures

Each experimental phase ran in multiple lab sessions with 1 to 6 participants per session. Lab sessions lasted approximately 1½h and involved participants completing a series of tasks. Following the review and completion of the study's consent document, each participant received a set of written instructions specific to the tasks dedicated to that study phase and lab session. To maintain a degree of uniformity, all participants who signed up for a given lab session participated in the same experimental phase. The experimenter facilitating the lab session read the instructions for the tasks aloud, making sure to provide ample opportunity to answer any possible questions. When all participants confirmed that they sufficiently understood the directions for the tasks that they were about to undertake, the experimenter guided participants to the start of the experiment, which took place via a computer.

Participants' initial involvement required them to complete a series of demographic questions presented on the computer screen. When participants had completed this portion of the experiment, they clicked on an icon labeled NEXT which then directed each participant to a screen that contained a brief instructional reminder for the upcoming task. After all participants indicated that they were ready to begin, they then clicked on the START icon displayed on the computer screen. The task that followed depended on the experimental phase that a participant was involved in during his or her lab session. The task of evaluating images for either mystery or fascination took between 20 and 30 min for participants to complete. Participants who carried out the RMT generally required 10–20 min for completion. Upon finishing one of these three opening tasks, the research team then provided participants with a short 2–3 min rest break.

Following the provided rest break, all participants regardless of their study phase, carried out the Aospan task. Completion of this task took participants between 15 and 20 min, after which the research team provided participants with another short 2–3 min rest break. With the conclusion of the second rest break, all participants completed the final two tasks of the lab session. The first was the DES, facilitated as a paper and pencil questionnaire. The second task was the English Fluency and Task questionnaire. Although not a cognitively based task, the questionnaire provided a means by which to review questionable or confounding data that might have resulted from language differences or from a participant having previous experience with the tasks used in the study. These final two tasks in the lab session took approximately 10 min or less to complete each. All lab sessions concluded with a short debriefing that aimed to provide participants with a more detailed explanation of the intentions for the study.

### Data analysis

In order to address each of the stated hypotheses, we conducted two primary sets of analyses. The first analysis involved conducting a Two-Way repeated-measures ANOVA in order to assess the effect that scene type (within-subjects factor) and presentation duration (between- subjects factor) had on a person's recognition memory performance. Results obtained from that analysis offered insight into whether images from a particular scene type evoked more automatic forms of processing. A comparison of participants' recognition performance for each scene type across all durations allowed us to better evaluate which durations to test for mediation; only those durations where participants' recognition performance was significantly different among scene types were tested.

Prior to testing for mediation it was necessary to first collapse the data garnered from the RMT down to a level that was consistent across each of the other two phases of the study. Although each phase of the study utilized a different group of participants, participants viewed the same images. As a result, testing for mediation at the image level was the most logical approach for data analysis. Examining data at the image level required calculating the mean recognition performance score for each of the 80 test images in the RMT (i.e., an items-analysis, collapsed across subject responses). To calculate that mean, data obtained from the RMT (Phase 2) was arranged in a manner that treated participants as variables and each image as a single observation^1^.

**Figure 1:**
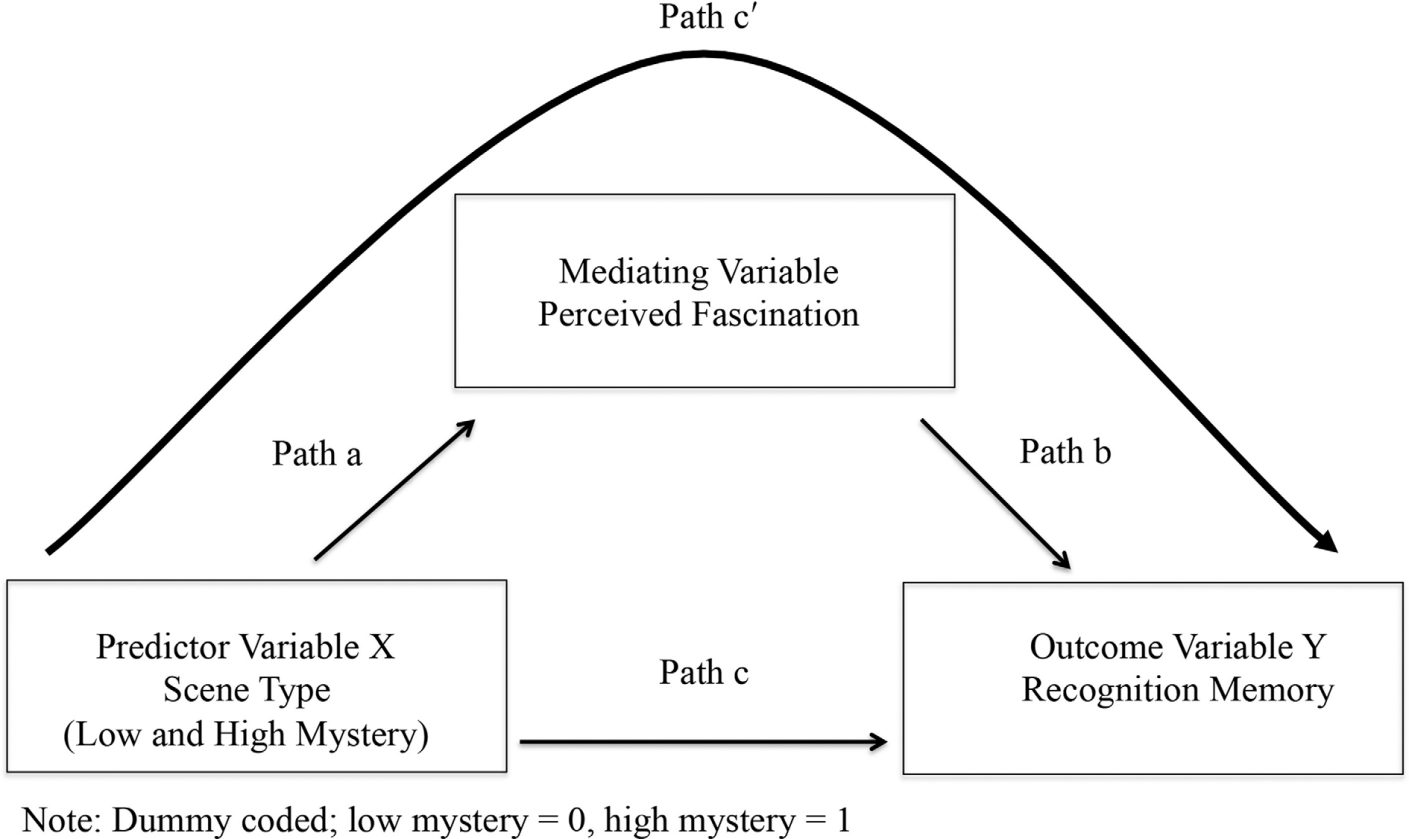
**Diagram of complete mediation model**.

Testing for mediation followed a three-step process that involved running a series of regression equations (Baron and Kenny, 1986; Kenny et al., 1998). Figure 1 presents the mediation model used for this study. In the first step, recognition memory performance served as the criterion variable with scene type as the predictor (Path c). This regression analysis aimed to establish a total effect between scene type and the mean rate of recognition memory performance for each of the 80 test images. In the second step, scores for fascination served as the criterion variable with scene type as the predictor (Path a). In order for fascination to be a mediating variable, variation in scene type had to significantly account for variation in scores for fascination. In the third and final step, recognition memory performance served as the criterion variable with both scene type (Path c) and perceived fascination as predictors (Path b). The function of this final regression analysis was to estimate the effect of scene type on the mean rate of recognition memory performance for each image (Path c′), when controlling for scores of fascination. If fascination fully or partially mediated the relationship between scene type and recognition memory, then the link between scene type and recognition memory would disappear or diminish as part of this regression analysis.

## Results

### Manipulation check

Given that individual differences in attention could have played a role in participants' ability to carry out certain tasks in the study, we attempted to ensure that there were minimal between group differences, and that groups were as homogenous as possible. As shown in Table 1, participants' total Aospan scores were relatively comparable across each phase of the study and consistent with scores found in similar works (Unsworth et al., 2005). Results from a One-Way ANOVA confirmed these initial findings as no significant differences were found between participants' total Aospan scores based on study phase, *F*_(2, 229)_ = 1.86, *p* = 0.157. In reviewing participants' scores on the DES, participants seemed to respond to the items of this scale in a similar fashion across each phase of the study (see Table 1). Results from a One-Way ANOVA again indicated that there were no significant differences between participants' average DES score, *F*_(2, 229)_ = 1.78, *p* = 0.170, based on study phase.

**Table 1:** **Distribution of Individual Differences**.

	**Mean**	**Std**.	**Skewness**	**Kurtosis**
**Phase 1 (*n* = 38)**				
AOSPAN	59.82	9.57	−0.101	−0.847
DES	1.85	0.324	0.398	−0.780
**Phase 2 (*n* = 144)**				
*300 ms (n = 36)*				
AOSPAN	56.28	13.58	−0.950	0.092
DES	1.72	0.448	1.57	3.55
*1 s (n = 36)*				
AOSPAN	53.69	17.81	−1.40	1.39
DES	1.92	0.432	0.915	0.835
*5 s (n = 36)*				
AOSPAN	52.91	28.03	−0.952	0.898
DES	1.99	0.497	0.708	−0.099
*10 s (n =36)*				
AOSPAN	59.19	11.82	−1.56	3.25
DES	1.94	0.427	1.49	3.34
**Phase 3 (*n* =**47**)**				
AOPSAN	52.98	13.40	−0.936	0.898
DES	1.76	0.368	1.06	2.70

### Mystery rating (phase 1)

**Figure 2:**
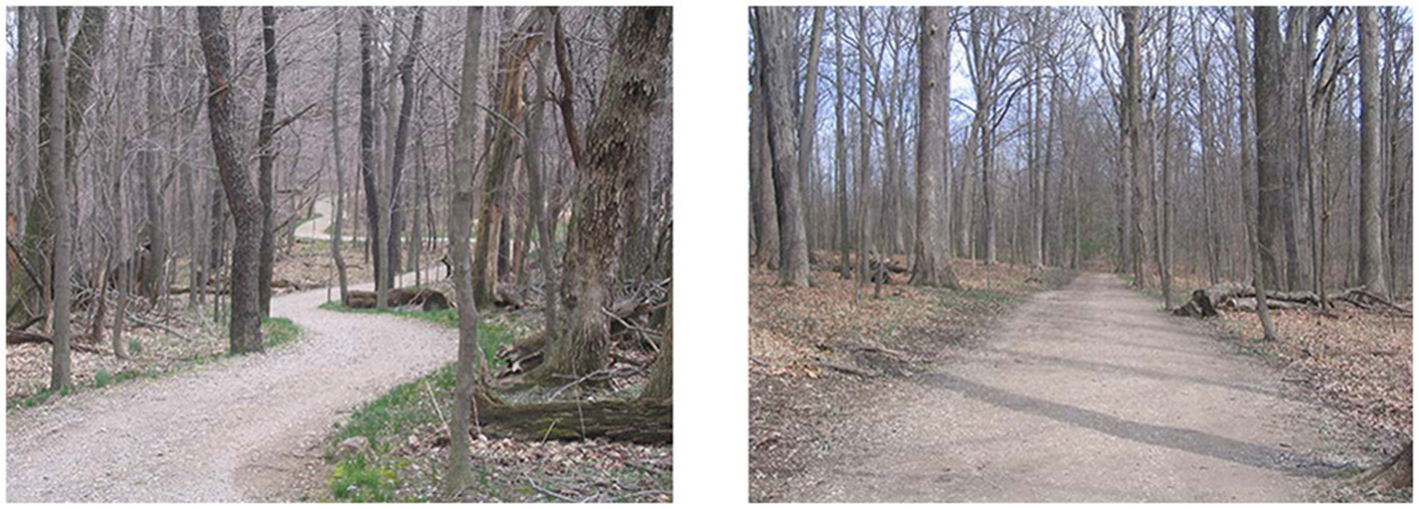
**Example of a high mystery (left) and low mystery (right) image**.

Results from Phase 1 appeared in the form of point ratings for mystery for each image rated by a participant. The analysis of those results involved examining the arithmetic means and standard deviations for each image through rank order. Standard deviations served as a measure of consensus among participants. Data analysis resulted in the selection of 80 images, 40 of which represented the highest ranked images (high mystery; *M* = 4.02, *SD* = 0.30), and 40 of which represented the lowest ranked images (low mystery; *M* = 2.51, *SD* = 0.165)^2^. Overall, response ratings derived from Phase 1 ranged on average from 1.89 to 4.89. As an additional manipulation check, an independent *t*-test demonstrated that images selected and grouped as low mystery and high mystery were statistically different *t*_(78)_ = 7.77, *p* < 0.05 in terms of their provided mean response rating. Results garnered from the following scenic assessment seemed to suggest and support the notion that mystery was a perceivable attribute in the trail scenes used for the present study (see Figure 2).

### Recognition memory task (phase 2)

#### Hit and false alarm rates for RMT

**Figure 3:**
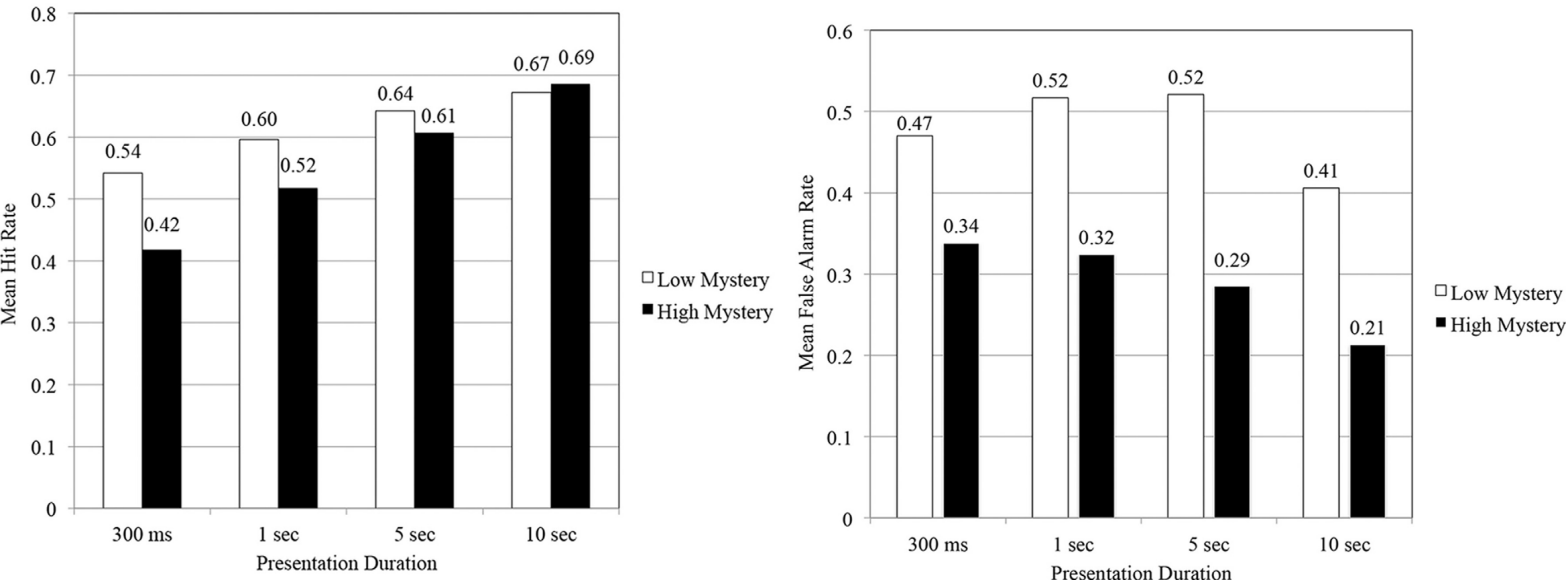
**Mean hit rates and false alarm rates as a function of scene type and presentation duration**.

Although rates of corrected recognition served as the criterion of interest in testing most of the study's hypotheses, analyses of hit and false alarm rates for the RMT offered a unique perspective from which to understand the contributions each factor played in recognition performance. As shown in Figure 3, participants correctly identified low mystery images seen at study more often than high mystery images for all durations except the 10 s duration. When examining changes in hit rate across time, however, the percentage of increase in hit rate for low mystery images from 300 ms to 10 s was 25%, whereas the percentage of increase among high mystery images for the same durations was 65%.

A repeated measures ANOVA showed a significant main effect for scene type, *F*_(1, 140)_ = 11.74, *p* < 0.01, η^2^ = 0.08. As well, that analysis indicated the presence of an interaction effect for scene type and presentation duration, *F*_(1, 140)_ = 3.29, *p* < 0.05, η^2^ = 0.07. An examination of simple main effects showed that participants' hit rate varied significantly for the 300 ms duration, *F*_(1, 35)_ = 8.84, *p* < 0.05, η^2^ = 0.20, and 1 s duration, *F*_(1, 35)_ = 6.67, *p* < 0.05, η^2^ = 0.16. Significant differences in hit rate did not occur at the 5 s duration, *F*_(1, 35)_ = 1.13, *p* = 0.295, η^2^ = 0.03, or 10 s duration, *F*_(1, 35)_ = 0.381, *p* = 0.541, η^2^ = 0.01. In these later durations, it would appear that images perceived high in mystery benefited more from the additional time given to study images as part of the RMT.

A review of false alarm rates offered some additional insight into the effect that scene type and presentation duration had on recognition performance. The data obtained from the RMT demonstrated that while low mystery images garnered a rather high hit rate, the false alarm rate for these images was also rather high (see Figure 3). Thus, it would appear that participants exercised a fairly liberal response pattern for low mystery images regardless if they had seen those images at study or not. Among images perceived high in mystery, participants were far less apt to erroneously identify images seen at study as “NEW,” especially when given more time to study those images. Similar response patterns often occur for low frequency words such as “silo” or “loft” (Otani and Whiteman, 1993). High mystery images, specifically high images never before seen, seemingly stood out, making it easier to discriminate between studied and non-studied images. This finding was reinforced as data denoted a significant main effect for scene type, *F*_(1, 140)_ = 117.76, *p* < 0.001, partial η^2^ = 0.45.

Further review of false alarm data revealed that an interaction effect was not present, *F*_(1, 140)_ = 1.46, *p* = 0.229, partial η^2^ = 0.03. A test of between subject effects indicated that false alarm rates varied significantly based on the duration of stimulus presentation, *F*_(3, 140)_ = 3.94, *p* < 0.05, η^2^ = 0.08. That is, with additional study time, there was a general reduction in participants' false alarm rate, with the most notable reduction in false alarms emerging at the longest duration (10 s).

#### Hypothesis 1

**Figure 4:**
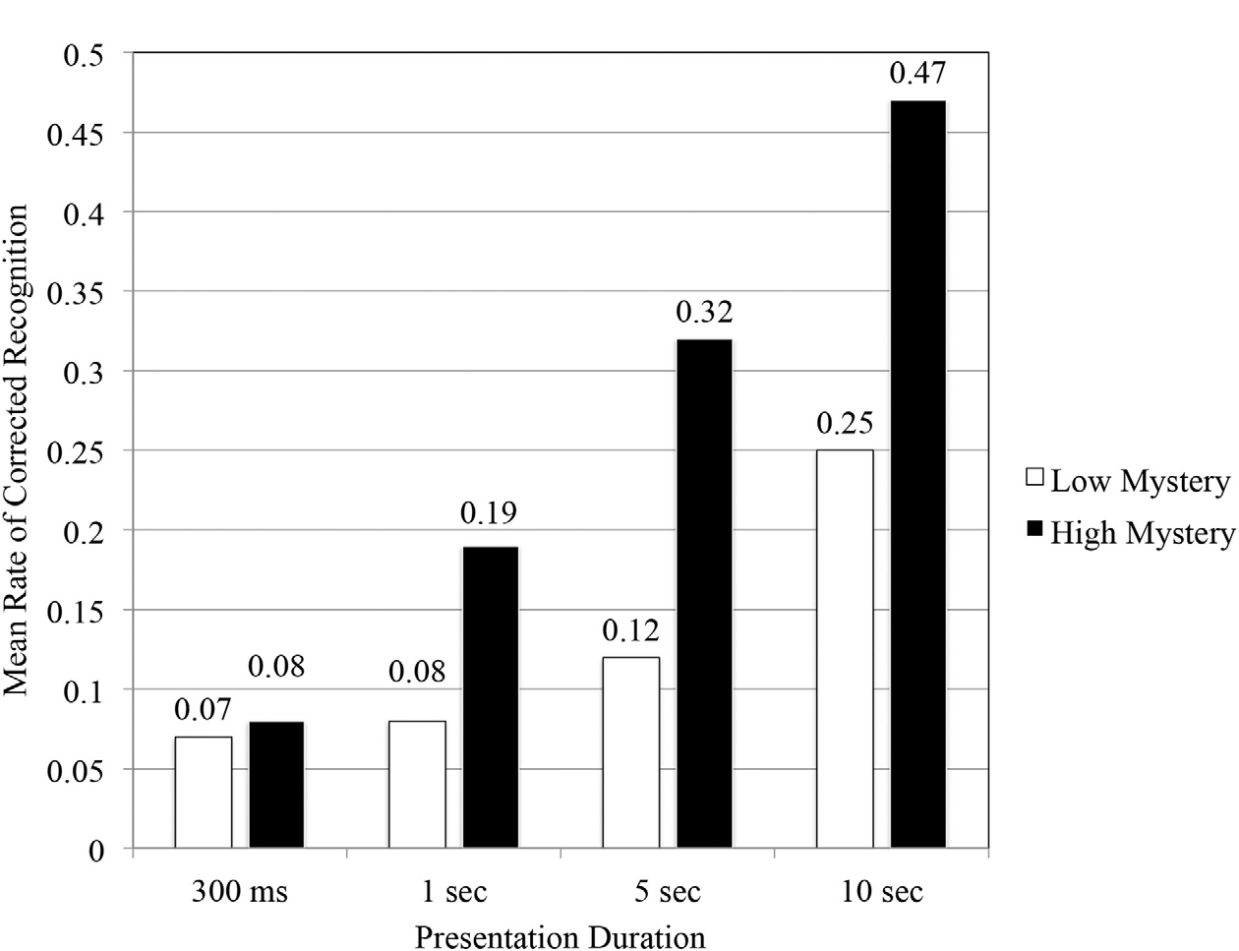
**Mean rate of corrected recognition as function of scene type and presentation duration**.

A review of participants' corrected recognition performance scores across all durations (see Figure 4) illustrated that under the proxy of time, high mystery images achieved a greater level of performance in a shorter period of time when compared to low mystery images. The results of the Two-Way repeated measures ANOVA indicated a significant main effect for scene type, *F*_(1, 140)_ = 41.27, *p* < 0.05, η^2^ = 0.23. As predicted (Hypothesis 1), that analysis also revealed a significant interaction effect for scene type and presentation duration, *F*_(3, 140)_ = 4.99, *p* < 0.05, η^2^ = 0.10. In order to better understand the nature of that interaction, an examination of simple main effects followed. At the fastest of the four durations, 300 ms, participants' corrected recognition performance did not vary significantly based on scene type, *F*_(1, 35)_ = 0.031, *p* = 0.861, η^2^ = 0.001. Study findings, however, did indicate that participants' corrected rate of recognition performance varied significantly based on scene type for the 1 s *F*_(1, 35)_ = 6.12, *p* < 0.05, η^2^ = 0.15, 5 s *F*_(1, 35)_ = 44.92, *p* < 0.001, η^2^ = 0.56, and 10 s *F*_(1, 35)_ = 51.84, *p* < 0.001, η^2^ = 0.60, durations. Under these conditions, images perceived high in mystery conveyed the greatest benefit in terms of performance on the RMT. As a result, tests for mediation focused solely on these specific durations where statistical differences for scene type existed.

#### Response rates for remember-know judgments

An examination of Remember (R) and Know (K) response rates provided an additional measure from which to understand the effect that scene type and presentation duration had on participants' recognition memory (see Table 2). In reviewing the presented R and K response probabilities, one can better assess the composition of recognition decisions. That is, the sum of the response rates for R and K judgments equals the response rates for recognition hits or false alarms, respectively, for a given scene type and duration. In the present study, there are certain response patterns that are noteworthy.

**Table 2:** **Response probability as a function of presentation duration and scene type**.

	**Hits**		**False alarms**	
	**Low mystery**	**High mystery**	**Low mystery**	**High mystery**
300 ms				
Remember	0.207	0.204	0.158	0.140
Know	0.335	0.214	0.311	0.189
1 s				
Remember	0.292	0.269	0.189	0.154
Know	0.304	0.249	0.328	0.168
5 s				
Remember	0.340	0.351	0.200	0.119
Know	0.310	0.258	0.307	0.165
10 s				
Remember	0.356	0.478	0.122	0.068
Know	0.338	0.213	0.286	0.149

Among high mystery images seen at study, the rate of R (0.204) and K (0.214) responses at the 300 ms duration were fairly comparable. As the amount of study time increased (10 s), however, participants were more likely to provide an R response (0.478), compared to a K response (0.213). Although a similar pattern appeared for low mystery images, the distinction between R and K responses was not as pronounced as it was for high mystery images. Results from a repeated measures ANOVA indicated a significant interaction effect among R responses for studied images based on scene type and presentation duration, *F*_(3,140)_ = 4.38, *p* < 0.01, η^2^ = 0.08. Simple main effects revealed that there was no difference in R response rates at the 300 ms duration, *F*_(1, 35)_ = 0.008, *p* = 0.928, η^2^ = 0.00, 1 s duration, *F*_(1, 35)_ = 0.790, *p* = 0.380, η^2^ = 0.02, or 5 s duration, *F*_(1, 35)_ = 0.079, *p* = 0.780, η^2^ = 0.00. At the 10 s duration, however, simple main effects indicated significant differences in recollection responses among studied images based on scene type, *F*_(1, 35)_ = 20.98, *p* < 0.001, η^2^ = 0.38 Drawing on these results, it appears that with additional time to study an image, participants had a far easier time recollecting certain contextual details for images perceived high in mystery compared to images perceived low in mystery.

Similar to recognition decisions, a review of R and K false alarm rates provides a more complete picture from which to assess the pattern of responses made by participants. The rate of false alarms for R and K responses is consistent with earlier findings obtained in our evaluation of participants' recognition performance (see Table 2). That is, images perceived high in mystery seemed to offer participants certain discriminative benefits over low mystery images. In general, participants were less likely to inaccurately identify a high mystery image as one that they could recollect with some detail. Results obtained from conducting a repeated measures ANOVA supported this notion as a evidence indicated a significant main effect for scene type, *F*_(1, 140)_ = 18.35, *p* < 0.001, η^2^ = 0.12. Results from this analysis did not, however, reveal the presence of an interaction effect, *F*_(3, 140)_ = 1.28, *p* = 0.282, η^2^ = 0.03. When viewed in conjunction with K false alarms, there would seem to be evidence that speaks to the liberal response pattern found in participants' rates of recognition performance for low mystery images. At the longer durations, images perceived low in mystery still elicited higher rates of K responses (5 s = 0.307, 10 s = 0.286). Thus, participants appeared to have a harder time distinguishing between studied and non-studied low mystery images.

**Figure 5:**
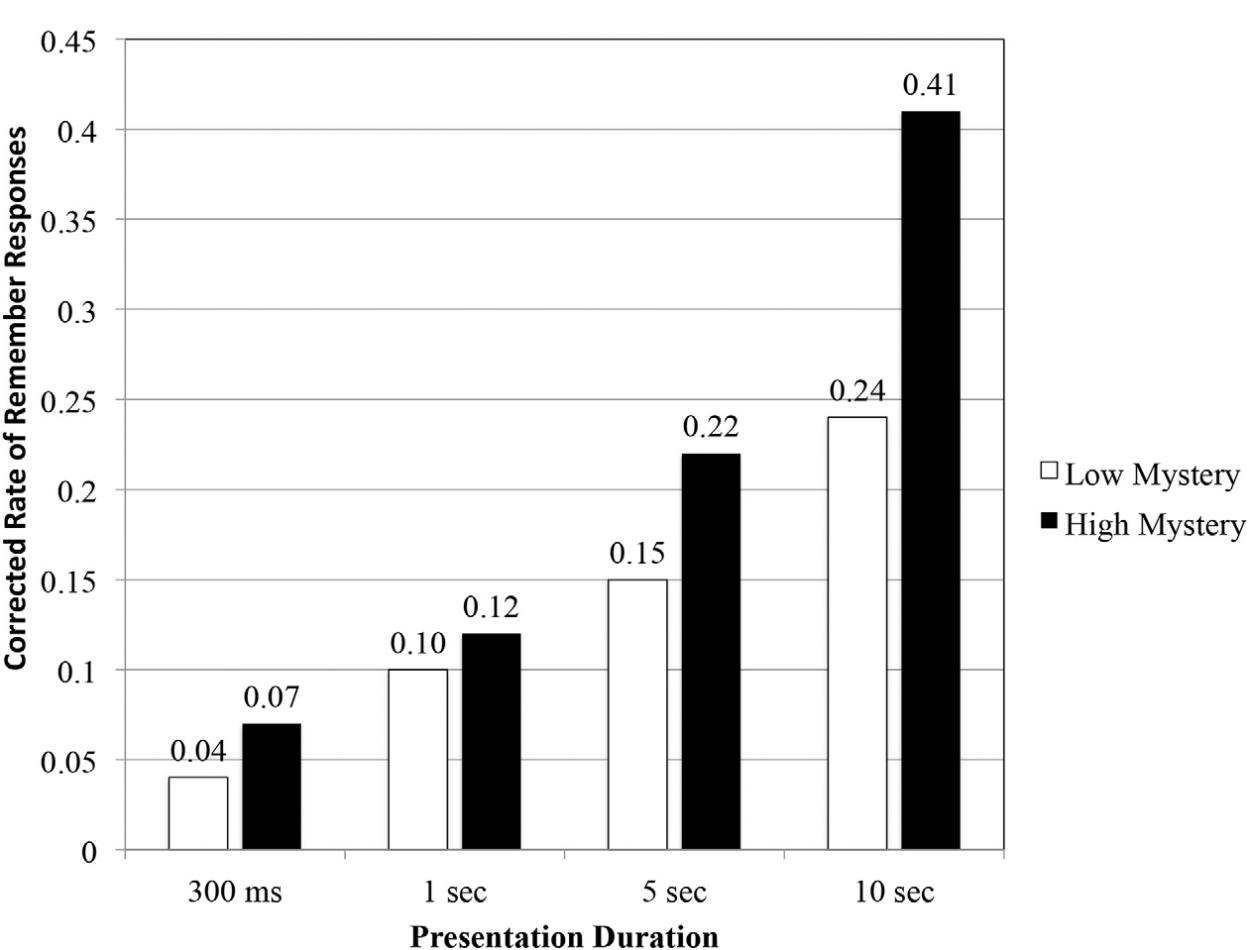
**Corrected rate of remember responses as a function of scene type and presentation duration**.

As a final appraisal, we examined the proportion of R-K responses after correcting for hits and false alarms (see Figure 5). Initial findings suggested that overall, images perceived high in mystery were more likely to elicit R responses from participants (0.41), compared to low mystery images (0.24).

Results obtained from a repeated measures ANOVA indicated a significant interaction effect, *F*_(3, 140)_ = 5.50, *p* < 0.01, η^2^ = 0.11. Simple main effects showed no significant differences for the 300 ms duration, *F*_(1, 35)_ = 0.167, *p* = 0.686, η^2^ = 0.005, and 1 s duration, *F*_(1, 35)_ = 0.205, *p* = 0.654, η^2^ = 0.006. This was not the case at the two longer durations. Evidence obtained from the RMT indicated significant differences in participants' corrected rate of remember responses for the 5 s duration, *F*_(1, 35)_ = 5.70, *p* < 0.05, η^2^ = 0.14 and 10 s duration, *F*_(1, 35)_ = 34.93, *p* < 0.001, η^2^ = 0.50. With additional study time, participants were more likely to recollect the contextual details of high mystery images compared to those grouped as low mystery. The pattern of results obtained from the R-K procedure seems to follow the same trend found in participants' recognition performance.

### Perceived fascination (phase 3)

Prior to running tests for mediation, mean fascination ratings were calculated for each RMT test image presented in Phase 3. Calculating mean fascination ratings involved taking the sum average of participants' responses to the three fascination items presented for each test image. Results from an independent *t*-test indicated that fascination ratings for low mystery (*M* = 2.59, *SD* = 1.33) and high mystery (*M* = 3.11, *SD* = 1.24) images were statistically different, *t*_(78)_ = −6.708, *p* < 0.001. Such findings support the proposed link between mystery and fascination found within the literature (Kaplan, 1978). With each of the variables in the mediation model represented, examining the indirect effects that fascination had on recognition performance via scene type could then proceed.

### Testing for mediation

Tests for mediation provided an opportunity to assess if scores for fascination served as the generative mechanism through which scene type influenced recognition performance. In accordance to Kenny et al. (1998), mediation testing involved running a series of regression equations. These analyses focused on the relations between scene type and recognition performance (Hypothesis 2), scene type and perceived fascination (Hypothesis 3), as well as fascination's role in mediating the effect scene type had on recognition (Hypothesis 4). In order to carry out the required regression equations, we first calculated the mean hit rate, false alarm rate, and corrected recognition rate for each test image (*n* = 80). As a result, tests for mediation occurred as items-analysis of RMT performance. Results of the regression analyses follow below.

#### Hypothesis 2

Initial tests for mediation focused on the 1 s duration. Images presented at this duration were among the first to demonstrate differences in recognition performance based on the type of image viewed. Despite these differences, however, the effect of scene type on corrected recognition was not significant (*R* = 0.219, *p* < 0.051, β = 0.219, *t* = 1.99, *p* = 0.051). Without the presence of a total effect, further testing for mediation at this duration did not continue as outlined by Baron and Kenny (1986). Testing for mediation at the 5 and 10 s durations did, however, yield evidence that confirmed the presence of a total effect (Path c). Regression results indicated that scene type significantly predicted recognition performance scores at both the 5 s (*R* = 0.445, *p* < 0.001, β = 0.445, *t* = 4.38, *p* < 0.001) and 10 s durations (*R* = 0.425, *p* < 0.001, β = 0.425, *t* = 4.15, *p* < 0.001). As hypothesized, the direction of this relationship was positive in that images perceived high in mystery resulted in improved rates of recognition performance.

#### Hypothesis 3

Having met the first requirement for mediation as predicted, a second regression equation assessed the effect that scene type had on mean scores for fascination (Path a). If the cognitive effects of mystery in nature presumably evoke more bottom-up forms of processing, then images perceived high in mystery should yield elevated ratings for fascination. Consistent with the proposed hypothesis (H3), variations in scene type significantly accounted for variations in mean fascination scores (*R* = 0.605, *p* < 0.001, β = 0.605, *t* = 6.71, *p* < 0.001). That is, images perceived high in mystery tended to produce higher scores for fascination. Results obtained from this analysis fulfilled the second criterion for mediation; establishing a significant relationship between the predictor variable and the presumed mediating variable.

#### Hypothesis 4

In the third and final regression equation, scene type (Path c) and fascination (Path b) served as predictors to estimate the effect of scene type on corrected recognition when controlling for fascination (Path c′). If the cognitive benefits that mystery has on recognition performance occur via its effects on fascination, then the mystery-recognition relationship should statistically depend on the mystery-fascination relationship. With that in mind, if fascination is the sole mediating variable responsible for the effect that scene type has on recognition performance, then this effect should disappear when controlling for fascination. If fascination partially mediates the effect that scene type has on recognition, then the relationship between scene type and recognition should diminish but remain significant. Tests for mediation demonstrated that the relationship between scene type and corrected recognition when controlling for fascination was no longer significant at both the 5 s (*R* = 0.631, *p* < 0.001, β = 0.105, *t* = 0.942, *p* = 0.349) and 10 s durations (*R* = 0.531, *p* < 0.001, β = 0.184, *t* = 1.52, *p* = 0.134). This in turn suggests that the effect of scene type on recognition performance was almost fully mediated by differences in perceived fascination for natural scenes presented in the RMT. With significant reductions in Beta, tests for mediation demonstrated, as predicted, that fascination was indeed a potent mediating variable.

## Discussion

In the present study, we set out to examine the cognitive benefits that mystery in nature had on attention. Findings from the study revealed several interesting insights. First, recognition performance among images perceived high in mystery increased disproportionately more over time than images perceived low in mystery. That is, at the fastest duration (300 ms), recognition performance among high mystery and low mystery images was no different. With additional study time however, recognition performance increased for both scene types, but significantly more so for images of the high mystery variety. The evidence garnered here not only provides a more focused appraisal of the cognitive processes activated when viewing nature scenes containing mystery, but also hints at the restorative potential that these kinds of settings may possess.

Our second major finding centers on results we obtained in testing for mediation. Initial tests indicated that mystery served as a powerful predictor in recognition performance. In a separate but related test, the data also demonstrated that mystery served as an effective predictor for perceptions of fascination. That is, images perceived high in mystery tended to yield higher ratings of perceived fascination for nature scenes used in the test portion of the RMT. Having met these initial conditions, our final analysis confirmed that the effects of mystery on attention occurred almost fully through perceptions of fascination.

In the information that follows, we consider the implications that these findings may have on our understanding of the cognitive benefits derived from interacting with nature. Our initial discussion focuses on the results obtained from the RMT as rates of recognition performance hint at the activation of certain forms of cognitive processing. We then address the repercussions that this might have in our interpretation of fascination, a factor believed to have a central role in the restorative process.

The study proceeded with the expectation that images perceived high in mystery would offer certain benefits over low mystery images in terms of recognition. The underlying contention was that with the prospect of acquiring additional information, settings perceived high in mystery would likely be more apt to engage a person's interest, and thus better positioned to evoke a more effortless form of attention (Kaplan, 1978). A review of recognition performance rates at the 300 ms duration provided the best prospect to test this supposition. At that presentation rate, processing often reflects a more automatic or effortless response, and should function effectively in a high workload situation (Schneider and Chein, 2003). As previously stated, there was no difference in recognition performance between the two scene types at the fastest presentation duration. As well, rates of recognition performance at this duration were rather low. This would seem to suggest that the processing of images was more top-down oriented, and thus required some expenditure of effort.

From a theoretical perspective, ART may provide some explanation for this outcome. Although sources of interest or fascination play a critical role in the restorative process, they are not sufficient by themselves. ART posits that fascination is just one of four interrelated factors that is necessary for a person to achieve the rest required for attentional recovery (Hartig et al., 1997; Kaplan and Berman, 2010). Within the context of the RMT, experiencing a sense of being away, extent, or compatibility might have been particularly challenging, especially at the fastest duration. Images presented at 300 ms likely did not allow a person enough time to establish the cognitive distance needed to free oneself from the demands occupying the mind. Instead of allowing fascination to come into function, the demands of the task maintained their prominence as images presented at study appeared at a rapid rate in succession.

In order to reduce the demands on one's cognitive capacity, ART also suggests that experiences of fascination must reflect a more persistent quality (Kaplan, 1995). Opportunities for sustained fascination occur when a person interacts with a setting that provides some sense of extent (Kaplan, 2001). Settings of this sort tend to be comprised of features that are not only rich in their content, but possess some degree of coherence. Collectively, these qualities help to establish the sensation that a person is experiencing a “whole other world” (Kaplan, 1995, p. 173). With so much to see, natural settings that possess ample content and structure tend to allow a person to remain engaged (Kaplan and Kaplan, 1989). In presenting images at the fastest duration, there was likely not enough time for a person to readily organize or explore the elements of the immediately perceived image. Given that consideration, one might presume that there was little opportunity to capture fascination, let alone sustain it over time.

The most discernible explanation for the results obtained in the fastest duration could be that the RMT lacked a degree of compatibility. Settings that do not support a person's intentions tend to require a considerable amount of mental effort (Herzog et al., 2011). With the presentation of images occurring at a fast rate, the demands imposed by the environment (task) were likely too great for the intention of encoding images into memory. Realistically, however, there are few scenarios where a person's interaction with a natural setting is for a mere fraction of a second. More often than not, a person has ample time to both physically and perceptually explore his or her surroundings. Although there is a strong contention that no one setting can provide a person with a source of rest indefinitely, we might also contend that human-nature interactions must be of sufficient duration in order for a person to realize the benefits of that interaction. An examination of the RMT results obtained at longer durations offers some support for this notion.

Results from the RMT showed that as the amount of time to study an image increased, there were subsequent improvements in rates of recognition performance for both scene types. Naturally, we might expect to see such improvements with additional study time. Under these circumstances, the opportunity for a person to purposively direct attention in order to encode images into memory was greater. If all things were then equal, recognition performance for the two scene types would have increased at the same rate (Barrouillet et al., 2004). The data obtained in this study, however, revealed that images perceived high in mystery benefited disproportionately more so across time than low mystery images. These effects seemed particularly pronounced at the longest two presentation durations (i.e., 5 s, 10 s).

Drawing on these results, it would appear that under the right conditions, the presence of mystery in nature had an additive effect on recognition performance. The improvements in recognition performance garnered by high mystery images likely occurred not only because of the mental effort invested, but also because of the salience that features within these types of settings possessed. Attentional control in such instances tends to be more stimulus-driven, or bottom-up oriented (Posner, 1980; Jonides, 1981). This combination of both top-down and bottom-up forms of processing clearly yielded certain direct benefits. In the case of this experiment, participants did not have to spend as much time viewing images perceived high in mystery to obtain the same level of performance they achieved for low mystery images at longer durations. In essence, participants were able to get more of a return on their investment of attention for images perceived high in mystery. Although not characteristically automatic by cognitive standards, the attentional resonance between top-down and bottom-up forms of processing could perhaps give the illusion that a particular task was more effortless in variety.

As further illustration of this notion, a review of Remember response rates by participants provided some interesting points of observation. Within each of the presentation durations, high mystery images tended to elicit more Remember responses than images perceived low in mystery. In addition, the disparity between the Remember response rates for high and low mystery images became even more distinct as the time to study a given image increased. Results obtained from this assessment likely occurred due to the hidden views that were common to images perceived high in mystery. Views of this sort invite a person to look more carefully at their surroundings, perhaps activating a deeper level of processing.

Although previous research has suggested that deeper levels of processing reflect a greater exertion of mental effort (Dewhurst and Hitch, 1999), the data obtained here could offer an alternative perspective that addresses the activation of attention. As participants viewed images perceived high in mystery, the prominent features within these settings captured attention in a more bottom-up fashion. In turn, the engagement of a person's attention conceivably occurred more fully, allowing a person to encode specific details of high mystery images into memory with greater ease.

In an effort to not only examine the effect that mystery in nature had on attention, but also understand why that effect occurred, we carried out tests for mediation. The focus of those examinations revolved around the notion that fascination provides a basis for resting attention (Cimprich, 1992; Kaplan, 1995; Berman et al., 2008). Stimuli perceived as fascinating presumably evoke a form of attention that is more automatic or effortless (Kaplan, 1995; Berto et al., 2008). By avoiding instances that call on directed attention, a person can thus rest that capacity (Kaplan, 1995).

To investigate the extent to which fascination served as a mediating variable, data analysis occurred at the image or item level, as opposed to the participant level. This approach to data analysis was a result of using an independent sample for each study phase. Utilizing an independent sample for each study phase avoided potential tautological problems that might have occurred from having a single sample rate images for certain physical qualities and then testing that sample's recognition memory for the same images. Tests for mediation demonstrated that perceptions of fascination almost fully mediated the effects that scene type had on recognition performance for the two longest durations.

The importance of this finding is twofold. First, in testing for mediation we discovered that fascination was an effective mediator only at longer presentation durations. These results indicate and support our earlier assertion that human-nature interactions likely require sufficient time in order for a person to experience the benefits of those interactions. As a second point of interest, previous conceptualizations of fascination have at times held a position that treats this restorative factor as a form of effortless attention (Kaplan, 1995; Berto, 2005). Drawing on the data obtained from the RMT, the advantages offered by high mystery images were not a result of an absence of effort, but rather the kind of effort a person expended. Discussion around this idea speaks directly to a number of theoretical considerations specific to ART.

### Theoretical considerations

In terms of restorative factors, researchers have often thought that fascination plays a critical role in providing a person with the rest that is necessary for attentional recovery (Kaplan, 1995; Berto et al., 2010; Kaplan and Berman, 2010). The significance of this restorative factor stems from James' (1890) delineation between two mechanisms of attention, one of which James argued, did not require effort. Drawing on this notion of attention, Kaplan (1995) adopted the term fascination. Sources of fascination presumably evoke an effortless form of attention that allows directed attention, a capacity that does require effort, to rest (Staats et al., 2003; Felsten, 2009). The data garnered from this study demonstrated that mystery in nature yielded certain attentional advantages as part of carrying out a recognition memory task.

The advantages offered by nature scenes perceived high in mystery were not a result of an absence of effort, but rather the kind of effort a person expended. In viewing scenes that contained environmental patterns related to mystery, the evidence here suggests that this outcome is, in part, a result of the activation of both top-down and bottom-up forms processing. The attentional resonance experienced by a person could perhaps more accurately reflect the direct benefit that person gains from interacting with these types settings. Interacting with settings whereby the patterns of stimulation are both inherently interesting and also parallel to one's goals would seem to have certain advantages.

Consider the distinction often made between hard and soft forms of fascination (Herzog et al., 1997). Experiences of hard fascination tend to embody circumstances in which the stimuli in the environment are so intense they completely consume a person's attention, leaving little opportunity for contemplation. Soft fascination, on the other hand, reflects those circumstances in which the hold on a person's attention is more modest, so as to not preclude a person from thought (Berto et al., 2008). Implicit within this notion of fascination is the understanding that a person is capable of exercising some volition over where she or he may be directing focus. Discussion surrounding these views of fascination provides some perspective from which to interpret the notion of attentional resonance.

In today's society, there is a great deal of disparity between what a person deems important, and what they find inherently interesting. As a result of this difference, many of the tasks a person must perform in everyday life necessitate a form of attention that is more top-down oriented. That is, the processing of information is largely the result of a person allocating attentional resources that are of a limited capacity. On the opposite side of the spectrum, there are countless sources of fascination (e.g., television, smartphones) that can effortlessly capture our attention, but leave us unfilled. Interactions with natural settings, however, tend to offer an appropriate balance between fascinations that modestly capture our attention, and circumstances that still permit a person to act with some volition. In recognition of this notion, it would seem clear that restorative experiences necessitate opportunities where both top-down and bottom-up forms of processing are active.

### Conclusion

For many people, there is a great deal of enjoyment in discovering that which lies just beyond the realm of their immediate perception. Nature, in all its grandeur, tends to comprise many settings that possess such qualities. The findings obtained from this study indicated that mystery in nature offered certain attention-related benefits. As evidenced, the direct benefit of viewing nature scenes containing mystery was the activation of a less demanding form of attention, which appeared to occur through perceptions of fascination. Extensions to the present study can proceed in variety of directions. Given that this study did not specifically examine the extent to which mystery facilitated attentional recovery, the most logical next step would involve investigating the restorative effects that mystery in nature has on attention. Such efforts would offer the opportunity to evaluate whether memory performance served as a valid indicator for a scene's ability to restore attention. At the same time, these efforts would likely further enhance our understanding of mystery as a scenic quality in nature.

As a scenic quality, mystery comprises one of the four informational variables identified within Kaplan and Kaplan's (1982) environmental preference matrix. Variables such as complexity, coherence, and legibility play an equally important role in influencing a person's preference for an environment (Kaplan, 1987; Stamps, 2004). In fact, previous research has shown that each of the four informational variables, to an extent, correlate with each other (Herzog and Kropscott, 2004; Herzog and Bryce, 2007). In the case of mystery, physical attributes that frequently contribute to a person's perception of mystery (i.e., spatial definition, screening, depth of field etc.) may also enhance the degree to which a person perceives a setting as complex. This potentially unavoidable challenge presents some unique limitations in deciphering the specific influence mystery may have on attention. Future research may wish to explore and further tease out the nuances that contribute to a person's perception of mystery in nature. Efforts directed at this aim may offer a more clear understanding of the effect mystery has on attention.

To better understand the effect that mystery has on attention, focused efforts may seek to also compare mystery in nature with mystery in more urban settings. Previous work has shown that people's perception of mystery is not always favorable under certain conditions (Herzog and Bryce, 2007; Nasar and Jones, 1997). Understanding the influence of mystery in different domains could prove useful from a park planning and design perspective. Finally, with the findings derived from this study, future research might also investigate the role that attentional resonance, a combination or balance of top-down and bottom-up forms of processing, have in the restoration process. Such advancements will not only allow us to better characterize the quality of rest that is so central to the restorative process, but also compel us to uncover the many mysteries and benefits that nature can provide.

### Conflict of interest statement

The authors declare that the research was conducted in the absence of any commercial or financial relationships that could be construed as a potential conflict of interest.

## Footnotes

^1^Within a given duration, half of the participants (*n* = 18) viewed one subset of images (i.e., images 1–40; Subset A) at study while the other half viewed a different subset of images (i.e., images 41–80; Subset B) at study. Participants in “Subset A” provided the hit rate for images 1–40, and the false alarm rate for images 41–80. Participants in “Subset B” provided the hit rate for images 41–80, and the false alarm rate for images 1–40. To determine the mean hit rate or mean false alarm rate of a given image, one simply divided the total of number of hits or false alarms for a given image by the total number of participants within that specific subset. The resulting percentage represented the mean hit or false alarm rate for an image. Subtracting the mean hit rate of an image from the mean false alarm rate of the same image produced the mean rate of corrected recognition performance for that image.

^2^The complete RMT image set has been made available as a supplementary resource, as well normative data (i.e., means, standard deviations) for mystery and fascination ratings for corresponding images.

## References

[B1] Baron R. M. Kenny D. A. (1986). The moderator-mediator variable distinction in social psychological research: conceptual, strategic, and statistical considerations. J. Pers. 51, 1173–1182. 3806354 10.1037//0022-3514.51.6.1173

[B2] Barrouillet P. Bernardin S. Camos V. (2004). Time constraints and resource sharing in adult's working memory spans. J. Exp. Psychol. 133, 83–100. 10.1037/0096-3445.133.1.83 14979753

[B3] Berman M. G. Jonides J. Kaplan S. (2008). The cognitive benefits of interacting with nature. Psychol. Sci. 19, 1207–1212 10.1111/j.1467-9280.2008.02225.x 19121124

[B4] Bernstein E. M. Putnam F. W. (1986). Development, reliability, and validity of a dissociation scale. J. Nerv. Ment. Dis. 174, 727–735. 3783140 10.1097/00005053-198612000-00004

[B5] Berto R. (2005). Exposure to restorative environments helps restore attentional capacity. J. Environ. Psychol. 25, 249–259 10.1016/j.jenvp.2005.07.001

[B6] Berto R. Baroni M. R. Zainaghi A. Bettella S. (2010). An exploratory study of the effect of high and low fascination environments on attentional fatigue. J. Environ. Psychol. 30, 494–500 10.1016/j.jenvp.2009.12.002

[B7] Berto R. Massaccesi S. Pasini M. (2008). Do eye movements measured across high and low fascination differ? addressing Kaplan's fascination hypothesis. J. Environ. Psyhcol. 28, 185–191 10.1016/j.jenvp.2007.11.004

[B8] Cimprich B. (1992). A theoretical perspective on attention and patient education. Adv. Nurs. Sci. 14, 40–51. 1347983 10.1097/00012272-199203000-00007

[B9] Dewhurst S. A. Hitch G. J. (1999). Cognitive effort and recollective experience in recognition memory. Memory 7, 129–146. 10645376 10.1080/741944067

[B10] Felsten G. (2009). Where to take a study break on the college campus: an attention restoration theory perspective. J. Environ. Psychol. 29, 160–167 10.1016/j.jenvp.2008.11.006

[B11] Gimblett H. R. Itami R. M. Fitzgibbon J. E. (1985). Mystery in an information processing model of landscape preference. Landsc. J. 4, 87–95.

[B12] Hammitt W. E. (1980). Designing mystery into trail-landscape experiences. J. Interpretation 5, 16–19.

[B13] Hartig T. Evans G. W. Jamner L. D. Davis D. S. Garling T. (2003). Tracking restoration in natural and urban field studies. J. Environ. Psychol. 23, 109–123 10.1016/S0272-4944(02)00109-3

[B14a] Hartig T. Kaiser F. G. Bowler P. A. (1997). Further Development of a Measure of Perceived Environmental Restorativeness. Working Paper No. 5. Institute for Housing Research, Uppsala University, Gävle, Sweden.

[B14] Hartig T. Korpela K. M. Evans G. W. Garling T. (1996). Validation of a Measure of Perceived Environmental Restorativeness (Goteb. Psychol. Rep. 26.7). Göteborg: Department of Psychology, Göteborg University.

[B15] Hartig T. Staats H. (2006). The need for psychological restoration as a determinant of environmental preferences. J. Environ. Psychol. 26, 215–226 10.1016/j.jenvp.2006.07.007

[B16] Herzog T. Black A. M. Fountaine K. A. Knotts D. J. (1997). Reflection and attentional recovery as distinctive benefits of restorative environments. J. Environ. Psychol. 17, 165–170.

[B17] Herzog T. R. (2007). Mystery and preference in within-forest settings. Environ. Behav. 39, 779–796 10.1177/0013916506298796

[B18] Herzog T. R. Bryce A. G. (2007). Mystery and preference in within-forest settings. Environ. Behav. 39, 779–776 10.1177/0013916506298796

[B19] Herzog T. R. Hayes L. J. Applin R. C. Weatherly A. M. (2011). Incompatibility and mental fatigue. Environ. Behav. 43, 827–847 10.1177/0013916510383242

[B20] Herzog T. R. Kropscott L. S. (2004). Legibility, mystery, and visual access as predictors of preference and perceived danger in forest settings without pathways. Environ. Behav. 36, 659–677 10.1177/0013916504264138

[B21] James W. (1890). The Principles of Psychology. New York, NY: Dover Publications.

[B22] Jonides J. (1981). Voluntary versus automatic control over the mind's eye's movement, in Attention and Performance, 9th Edn., eds Long J. B. Baddeley A. D. (Hillsdale, NJ: Erlbaum), 187–203.

[B23] Kaplan R. Kaplan S. (1989). The Experience of Nature: A Psychological Perspective. New York, NY: Cambridge University Press.

[B24] Kaplan S. (1978). Attention and fascination: the search for cognitive clarity, in Humanscape: Environments for People, eds Kaplan S. Kaplan R. (Ann Arbor, MI: Ulrich's Books), 84–93.

[B25] Kaplan S. (1987). Aesthetics, affect, and cognition: environmental preference from an evolutionary perspective. Environ. Behav. 19, 3–32.

[B26] Kaplan S. (1992). The restorative environment: nature and human experience, in The Role of Horticulture in Human Well-being and Social Development, ed Relf D. (Portland, OR: Timber), 134–142.

[B27] Kaplan S. (1995). The restorative benefits of nature: toward an integrative framework. J. Environ. Psychol. 15, 269–182.

[B28] Kaplan S. (2001). Meditation, restoration, and the management of mental fatigue. Environ. Behav. 33, 480–506 10.1177/00139160121973106

[B29] Kaplan S. Berman M. G. (2010). Directed attention as a common resource for executive functioning and self-regulation. Perspect. Psychol. Sci. 5, 43–57 10.1177/1745691609356784 26162062

[B30] Kaplan S. Kaplan R. (1982). Cognition and Environment: Functioning in an Uncertain World. New York, NY: Praeger.

[B31] Kaplan S. Talbot J. F. (1983). Psychological benefits of a wilderness experience, in Human behavior and environment, Vol. 6, eds Altman I. Wohlwill J. F. (New York, NY: Plenum), 163–203.

[B32] Kenny D. A. Kashy D. A. Bolger N. (1998). Data analysis in social psychology, in The Handbook of Social Psychology, 4th Edn., Vol. 1, eds Gilbert D. Fiske S. T. Lindzey G. (New York, NY: McGraw-Hill), 223–265.

[B33] Kjellgren A. Buhrkall H. (2010). A comparison of the restorative effect of a natural environment with that of a simulated natural environment. J. Environ. Psyhcol. 30, 464–472 10.1016/j.jenvp.2010.01.011

[B34] Knopf R. C. (1987). Human behavior, cognition, and affect in the natural environment, in Handbook of Environmental Psychology, Vol. 1, eds Stokols D. Altman I. (New York, NY: Wiley), 783–825.

[B35] Kuo F. E. Sullivan W. C. (2001). Aggression and violence in the inner city: effects of environment via mental fatigue. Environ. Behav. 33, 543–571 10.1177/00139160121973124

[B36] Nasar J. L. Jones K. M. (1997). Landscapes of fear and stress. Environ. Behav. 29, 291–323.

[B37] Otani H. Whiteman H. L. (1993). Word frequency effect: a test of a processing-based explanation. Psychol. Rec. 43, 317–327.

[B38] Posner M. I. (1980). Orienting of attention, Q. J. Exp. Psychol. 32, 2–25. 10.1080/00335558008248231 7367577

[B39] Posner M. I. Snyder C. R. R. Davidson B. J. (1980). Attention and the detection of signals. J. Exp. Psychol. 109, 160–174. 7381367

[B40] Posner M. Snyder C. (1975). Attention and cognitive control, in Information Processing and Cognition, ed Solso R. L. (Hillsdale, NJ: Erlbaum), 55–85.

[B41] Schneider W. Chein J. M. (2003). Controlled and automatic processing: behavior, theory, and biological mechanisms. Cogn. Sci. 27, 529–559. 10.1016/S0364-0213(03)00011-918579344

[B42] Staats H. Kieviet A. Hartig T. (2003). Where to recover from attentional fatigue: an expectancy analysis of environmental preference. J. Environ. Psychol. 23, 147–157 10.1016/S0272-4944(02)00112-3

[B43] Stamps A. E. (2004). Mystery, complexity, legibility, coherence: a meta-analysis. J. Environ. Psychol. 24, 1–16 10.1016/S0272-4944(03)00023-9

[B44] Taylor A. F. Kuo F. E. (2009). Children with attention deficits concentrate better after a walk in the park. J. Atten. Disord. 12, 402–409. 10.1177/1087054708323000 18725656

[B45] Tennessen C. Cimprich B. (1995). Views to nature: effects on attention. J. Environ. Psychol. 15, 77–85.

[B46] Unsworth N. Heitz RP. Schrock J. C. Engle R. W. (2005). An automated version of the operation span task. Behav. Res. Methods 37, 498–505. 10.3758/BF03192720 16405146

[B47] Watson J. M. Balota D. A. Roediger I. I. I., H. L. (2003). Creating false memories with hybrid lists of semantic and phonological associates: over additive false memories produced by converging associative networks. J. Mem. Lang. 49, 95–118 10.1016/S0749-596X(03)00019-6

