# Supplementary material for: The benefits of mystery in nature on attention: assessing the impacts of presentation duration
Source: Front Psychol. 2014 Nov 25;5:1360. doi: 10.3389/fpsyg.2014.01360 (PMC4244865; doi:10.3389/fpsyg.2014.01360)
Supplement: Supplementary file 1 [file DataSheet1.PDF]

Mean Fascination Ratings – Low Mystery Images			Mean Fascination Ratings – High Mystery Images		
Image #	Mean Fascination Rating	SD	Image #	Mean Fascination Rating	SD
0_1	2.6950	1.56475	1_1	3.6738	1.32878
0_2	2.5248	1.66194	1_2	3.0000	1.62261
0_3	2.2979	1.49756	1_3	2.5248	1.66630
0_4	2.8014	1.55520	1_4	3.1915	1.44420
0_5	2.4113	1.52628	1_5	3.6525	1.41926
0_6	2.8794	1.69316	1_6	3.1064	1.65956
0_7	2.2979	1.46824	1_7	3.2340	1.41748
0_8	2.0780	1.58223	1_8	3.0780	1.49591
0_9	2.4043	1.53374	1_9	2.9149	1.54399
0_10	2.4823	1.66784	1_10	3.6950	1.57552
0_11	2.7305	1.54582	1_11	3.3617	1.48068
0_12	2.9645	1.56692	1_12	3.0426	1.50140
0_13	2.7943	1.63085	1_13	3.7305	1.67914
0_14	2.6454	1.50266	1_14	3.2411	1.46407
0_15	2.5390	1.48674	1_15	2.7872	1.63510
0_16	2.2766	1.55622	1_16	3.5390	1.48999
0_17	2.4184	1.45706	1_17	2.7730	1.50700
0_18	2.5035	1.47257	1_18	2.7376	1.38476
0_19	2.3972	1.61610	1_19	3.4894	1.35463
0_20	2.7730	1.58359	1_20	3.4610	1.33975
0_21	2.4043	1.54159	1_21	3.2837	1.41929
0_22	2.3972	1.69204	1_22	2.7730	1.54186
0_23	3.0142	1.46778	1_23	3.7872	1.54079
0_24	2.1560	1.52729	1_24	3.3475	1.54319
0_25	2.3972	1.65888	1_25	2.7801	1.56235
0_26	3.7234	1.47330	1_26	2.8298	1.45939
0_27	2.2908	1.60858	1_27	2.3475	1.59552
0_28	2.6099	1.50574	1_28	3.0355	1.53892
0_29	2.7589	1.45745	1_29	3.0000	1.57118
0_30	3.0213	1.68168	1_30	3.3050	1.69231
0_31	2.7730	1.49896	1_31	3.0922	1.39132
0_32	2.4894	1.52887	1_32	2.9291	1.48899
0_33	2.8369	1.41400	1_33	3.3333	1.39357
0_34	2.9291	1.50673	1_34	3.6879	1.27670
0_35	2.8014	1.59961	1_35	2.5674	1.66219
0_36	2.3688	1.55764	1_36	2.5816	1.49309

0_37	2.1560	1.53360	1_37	2.7943	1.54725
0_38	2.7872	1.49302	1_38	2.8440	1.61642
0_39	2.2128	1.55484	1_39	2.6525	1.40213
0_40	2.6454	1.73818	1_40	3.2411	1.61021
