# Supplementary material for: The benefits of mystery in nature on attention: assessing the impacts of presentation duration
Source: Front Psychol. 2014 Nov 25;5:1360. doi: 10.3389/fpsyg.2014.01360 (PMC4244865; doi:10.3389/fpsyg.2014.01360)
Supplement: Supplementary file 2 [file DataSheet2.PDF]

Perceived Low Mystery Images			Perceived High Mystery Images		
**Image #**	**Mean Mystery Rating**	**SD**	**Image #**	**Mean Mystery Rating**	**SD**
0_1	1.8947	1.68923	1_1	4.8947	1.06007
0_2	2.1579	1.76339	1_2	4.7895	1.23371
0_3	2.2632	1.30869	1_3	4.7632	1.32408
0_4	2.2632	1.32918	1_4	4.4211	1.13021
0_5	2.2895	1.91591	1_5	4.4211	1.19981
0_6	2.3158	1.61254	1_6	4.3158	1.1879
0_7	2.3158	1.33771	1_7	4.2895	1.39319
0_8	2.3421	1.38088	1_8	4.1842	1.20484
0_9	2.4737	1.38986	1_9	4.1842	1.2489
0_10	2.4737	1.42823	1_10	4.1316	1.18939
0_11	2.4737	1.79695	1_11	4.0789	1.2815
0_12	2.5	1.70452	1_12	4.0789	1.2386
0_13	2.5	1.53781	1_13	4.0526	1.4322
0_14	2.5263	1.48391	1_14	4.0526	1.37443
0_15	2.5263	1.50201	1_15	4.0263	1.3046
0_16	2.5263	1.48391	1_16	4.0263	1.17374
0_17	2.5526	1.8262	1_17	3.9737	1.21892
0_18	2.5526	2.5526	1_18	3.9737	1.3454
0_19	2.5526	1.48319	1_19	3.9737	1.3454
0_20	2.5526	1.24548	1_20	3.9211	1.30242
0_21	2.5526	1.44629	1_21	3.9211	1.04962
0_22	2.5526	1.5013	1_22	3.8947	1.46655
0_23	2.5526	1.5013	1_23	3.8947	1.75206
0_24	2.5789	1.24405	1_24	3.8947	1.31086
0_25	2.5789	1.3076	1_25	3.8947	1.35147
0_26	2.5789	1.57053	1_26	3.8947	1.00779
0_27	2.5789	1.46364	1_27	3.8684	1.25571
0_28	2.6053	1.34646	1_28	3.8684	1.2119
0_29	2.6053	1.26362	1_29	3.8421	1.48007
0_30	2.6053	1.82386	1_30	3.8421	1.19744
0_31	2.6053	1.60303	1_31	3.8158	1.52201
0_32	2.6053	1.60303	1_32	3.7895	1.35881
0_33	2.6316	1.60103	1_33	3.7895	1.35881
0_34	2.6316	1.58406	1_34	3.7632	1.21776
0_35	2.6316	1.58406	1_35	3.7632	1.3643
0_36	2.6579	1.45707	1_36	3.7368	1.201
0_37	2.6842	1.52644	1_37	3.7368	1.52294
0_38	2.6842	1.59569	1_38	3.7105	1.52297
0_39	2.6842	1.41622	1_39	3.71105	1.3133
0_40	2.7105	1.50509	1_40	3.6842	1.87595
