# Supplementary figures and images for: The benefits of mystery in nature on attention: assessing the impacts of presentation duration
Source: Front Psychol. 2014 Nov 25;5:1360. doi: 10.3389/fpsyg.2014.01360 (PMC4244865; doi:10.3389/fpsyg.2014.01360)

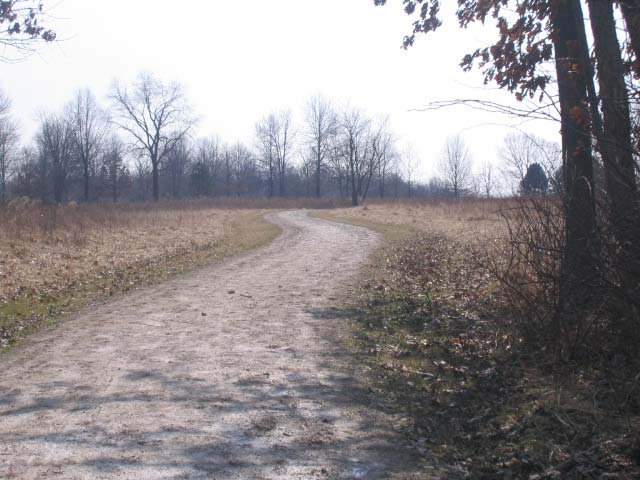

Supplement: Supplementary file 3 [file DataSheet3.ZIP › LowMystery/0_1.jpg]

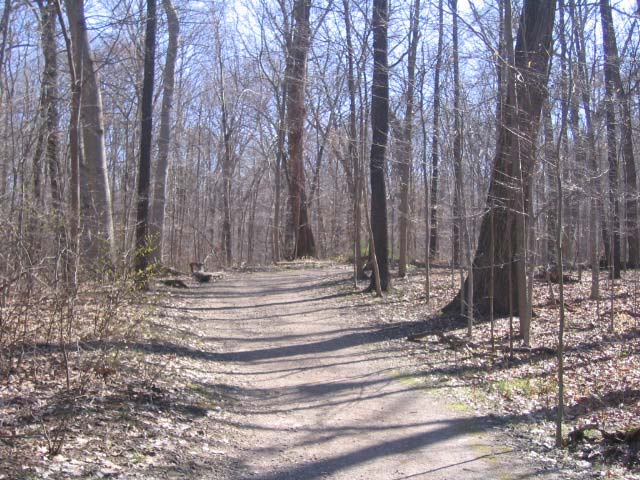

Supplement: Supplementary file 3 [file DataSheet3.ZIP › LowMystery/0_10.jpg]

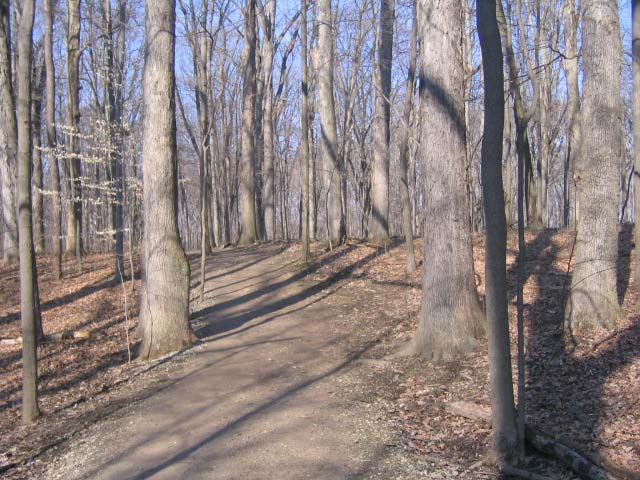

Supplement: Supplementary file 3 [file DataSheet3.ZIP › LowMystery/0_11.jpg]

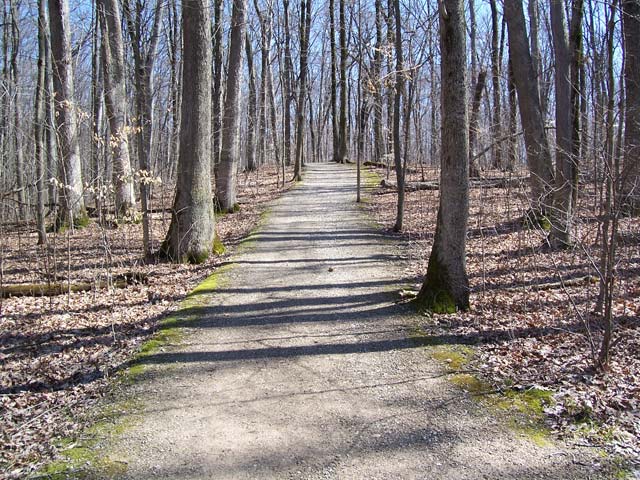

Supplement: Supplementary file 3 [file DataSheet3.ZIP › LowMystery/0_12.jpg]

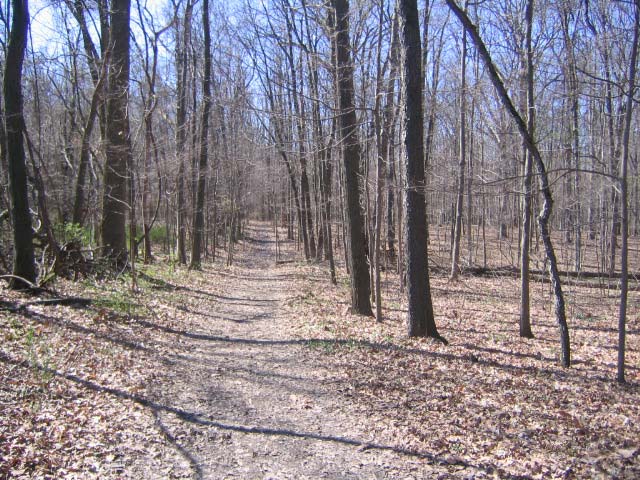

Supplement: Supplementary file 3 [file DataSheet3.ZIP › LowMystery/0_13.jpg]

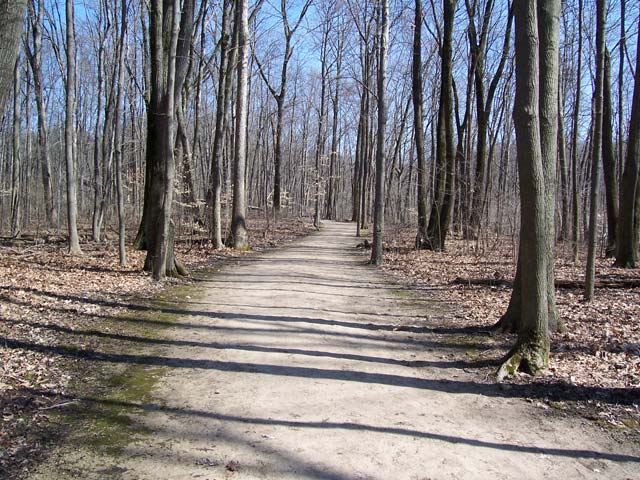

Supplement: Supplementary file 3 [file DataSheet3.ZIP › LowMystery/0_14.jpg]

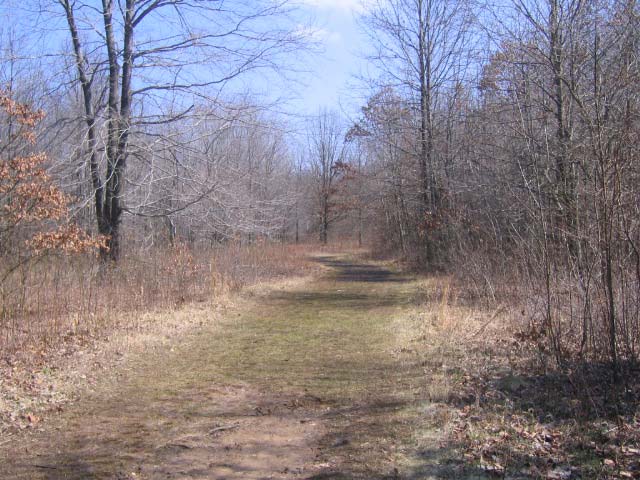

Supplement: Supplementary file 3 [file DataSheet3.ZIP › LowMystery/0_15.jpg]

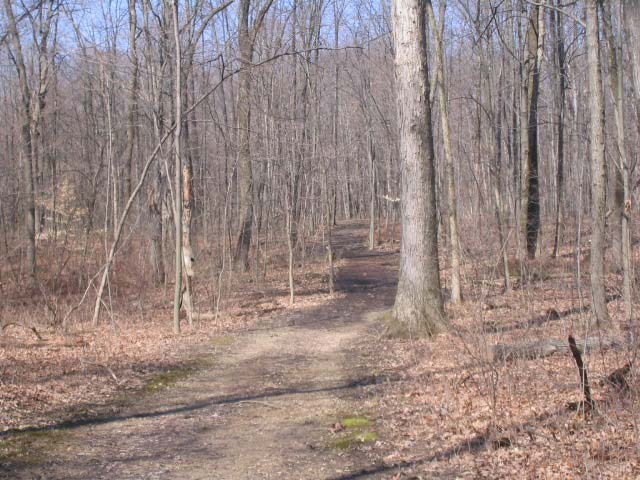

Supplement: Supplementary file 3 [file DataSheet3.ZIP › LowMystery/0_16.jpg]

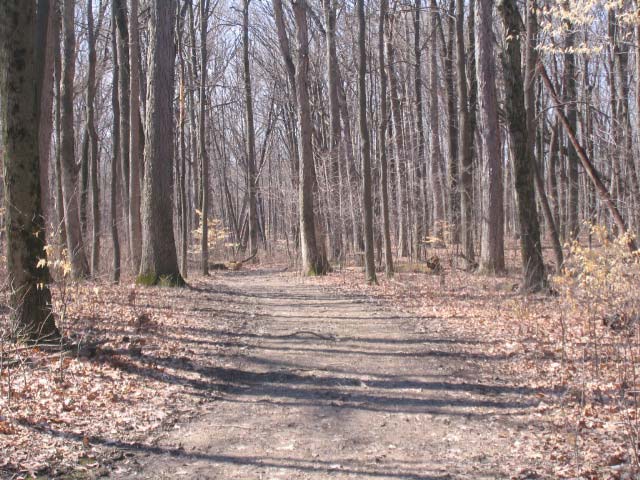

Supplement: Supplementary file 3 [file DataSheet3.ZIP › LowMystery/0_17.jpg]

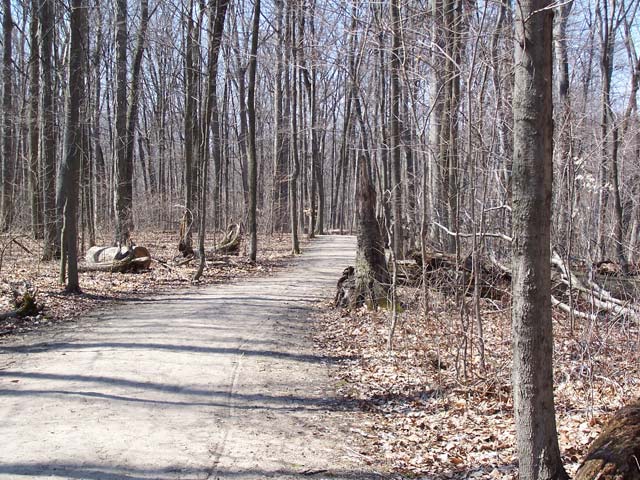

Supplement: Supplementary file 3 [file DataSheet3.ZIP › LowMystery/0_18.jpg]

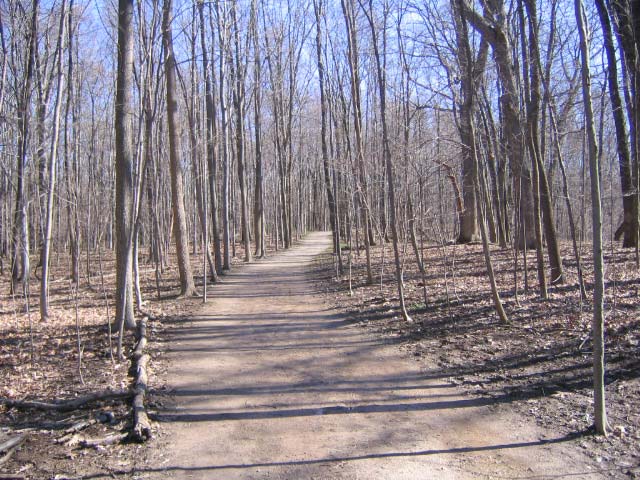

Supplement: Supplementary file 3 [file DataSheet3.ZIP › LowMystery/0_19.jpg]

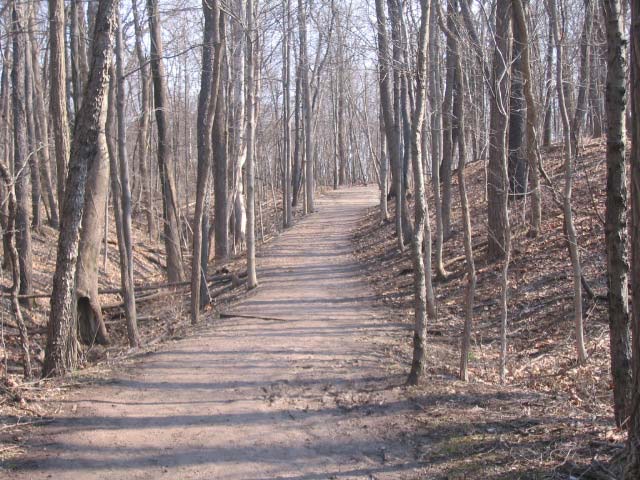

Supplement: Supplementary file 3 [file DataSheet3.ZIP › LowMystery/0_2.jpg]

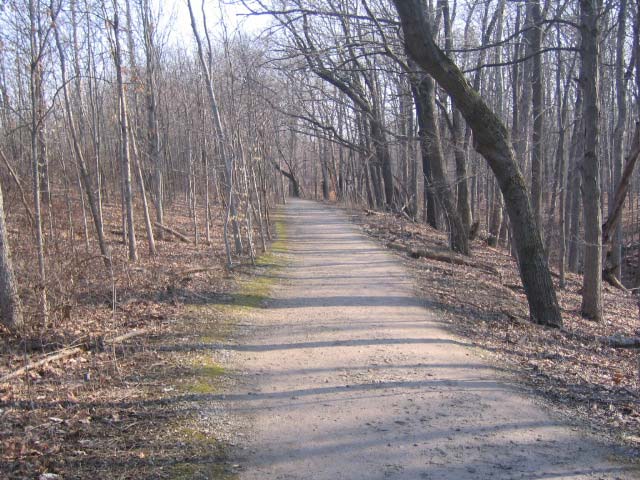

Supplement: Supplementary file 3 [file DataSheet3.ZIP › LowMystery/0_20.jpg]

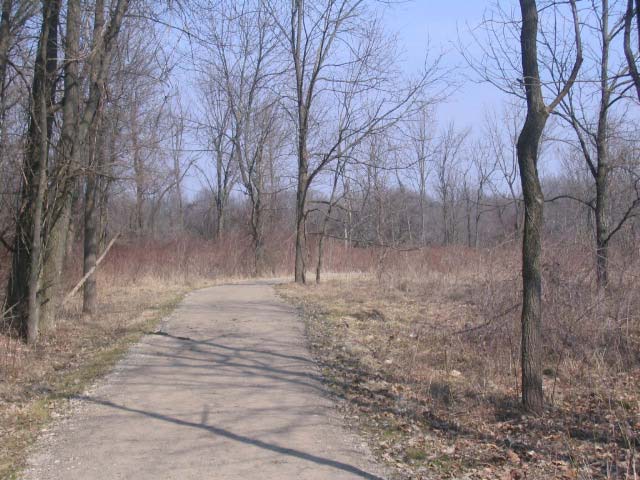

Supplement: Supplementary file 3 [file DataSheet3.ZIP › LowMystery/0_21.jpg]

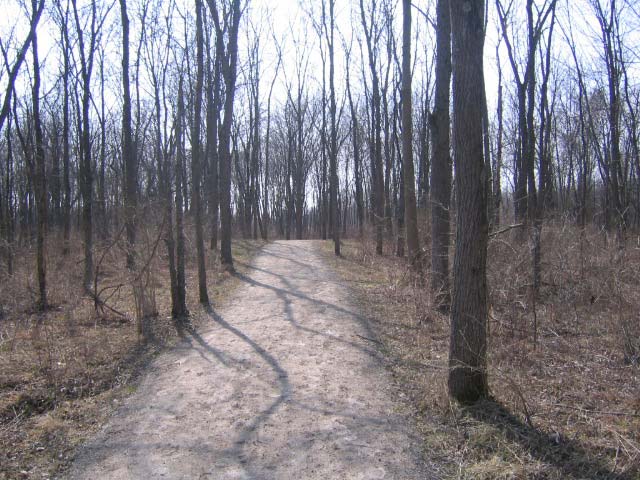

Supplement: Supplementary file 3 [file DataSheet3.ZIP › LowMystery/0_22.jpg]

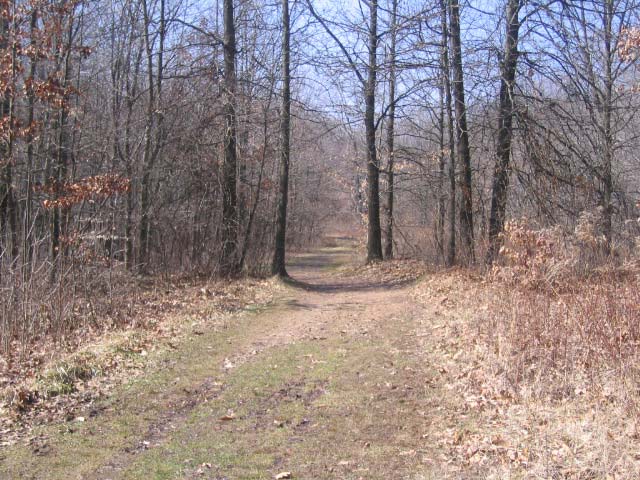

Supplement: Supplementary file 3 [file DataSheet3.ZIP › LowMystery/0_23.jpg]

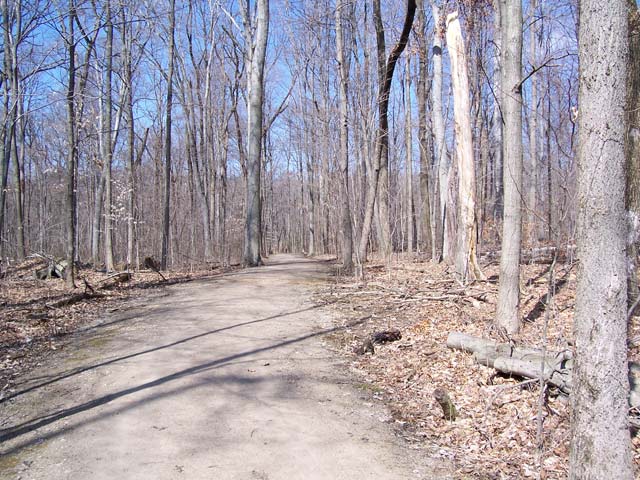

Supplement: Supplementary file 3 [file DataSheet3.ZIP › LowMystery/0_24.jpg]

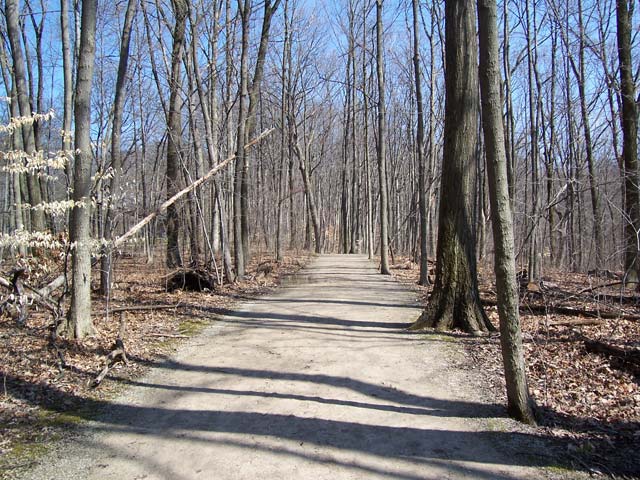

Supplement: Supplementary file 3 [file DataSheet3.ZIP › LowMystery/0_25.jpg]

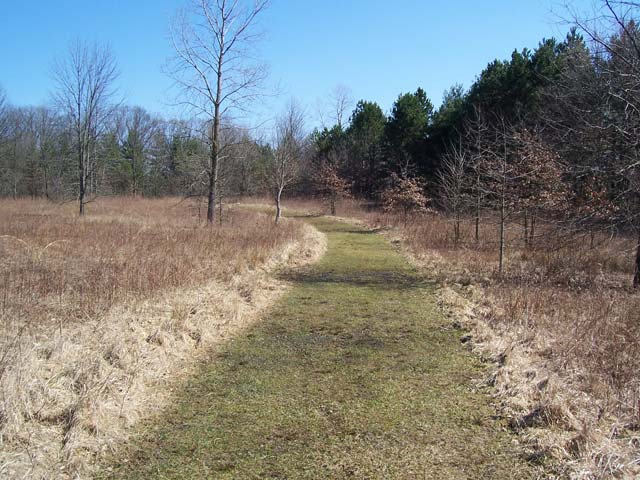

Supplement: Supplementary file 3 [file DataSheet3.ZIP › LowMystery/0_26.jpg]

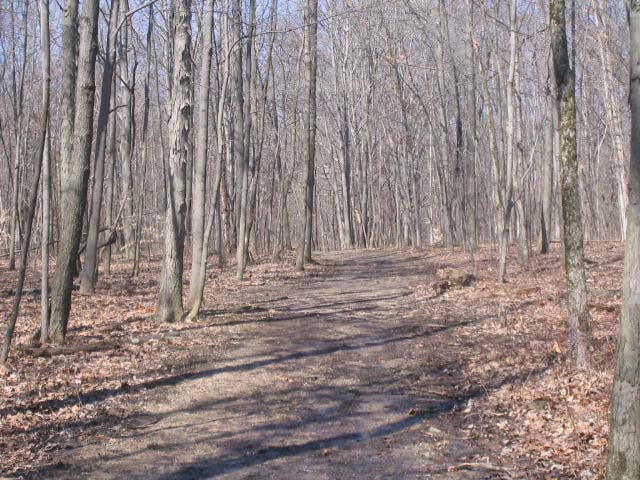

Supplement: Supplementary file 3 [file DataSheet3.ZIP › LowMystery/0_27.jpg]

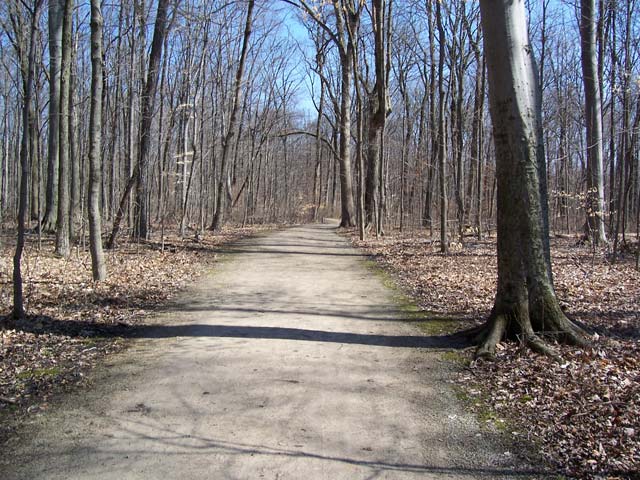

Supplement: Supplementary file 3 [file DataSheet3.ZIP › LowMystery/0_28.jpg]

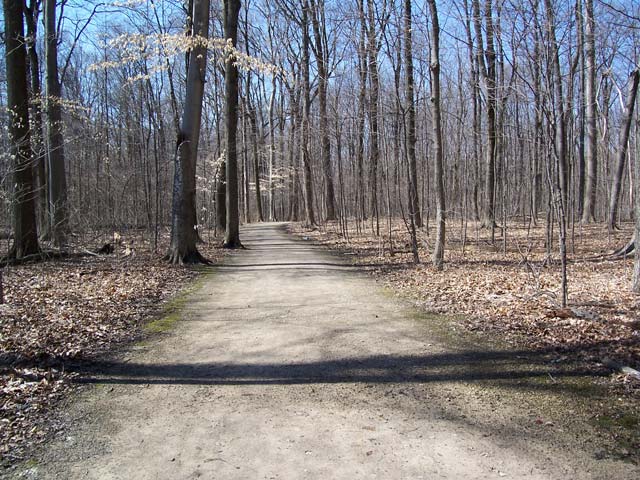

Supplement: Supplementary file 3 [file DataSheet3.ZIP › LowMystery/0_29.jpg]

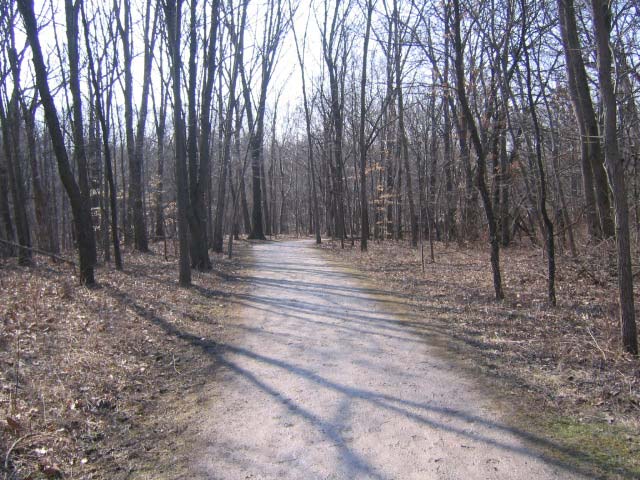

Supplement: Supplementary file 3 [file DataSheet3.ZIP › LowMystery/0_3.jpg]

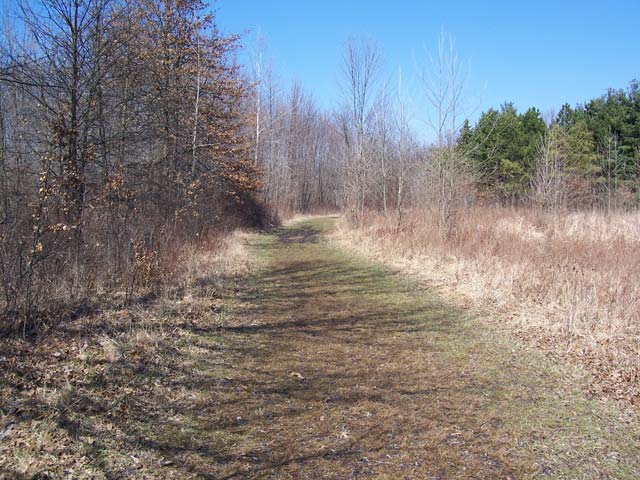

Supplement: Supplementary file 3 [file DataSheet3.ZIP › LowMystery/0_30.jpg]

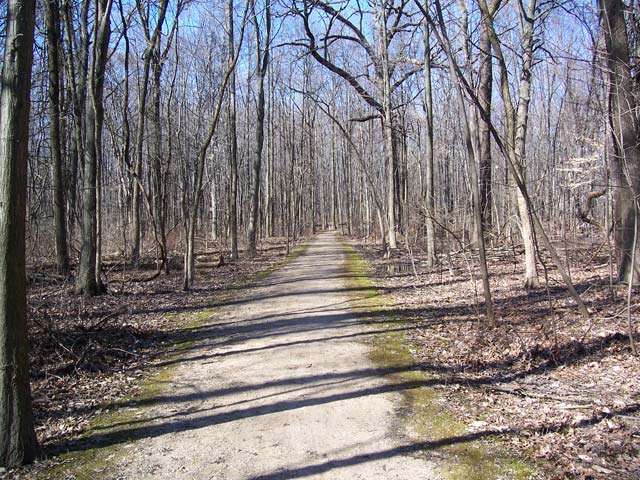

Supplement: Supplementary file 3 [file DataSheet3.ZIP › LowMystery/0_31.jpg]

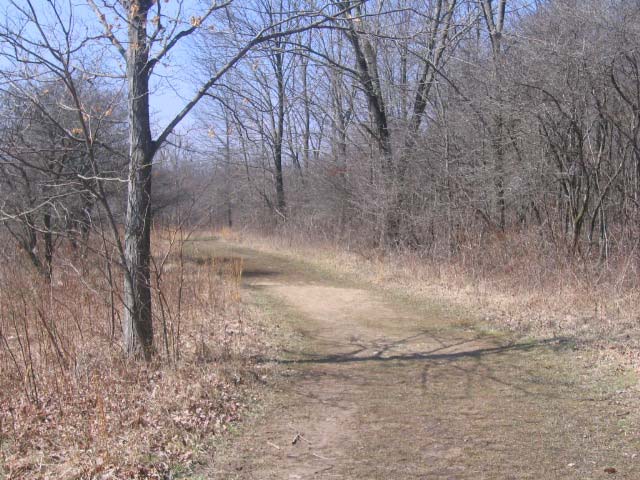

Supplement: Supplementary file 3 [file DataSheet3.ZIP › LowMystery/0_32.jpg]

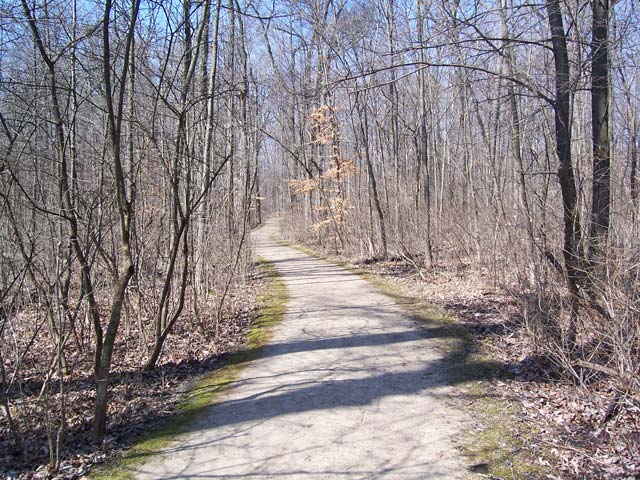

Supplement: Supplementary file 3 [file DataSheet3.ZIP › LowMystery/0_33.jpg]

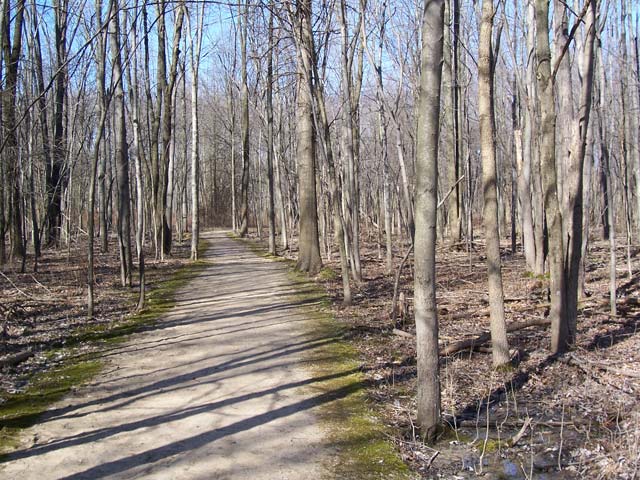

Supplement: Supplementary file 3 [file DataSheet3.ZIP › LowMystery/0_34.jpg]

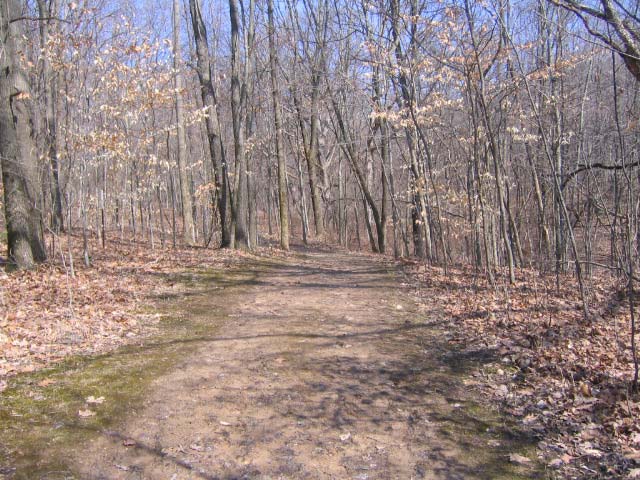

Supplement: Supplementary file 3 [file DataSheet3.ZIP › LowMystery/0_35.jpg]

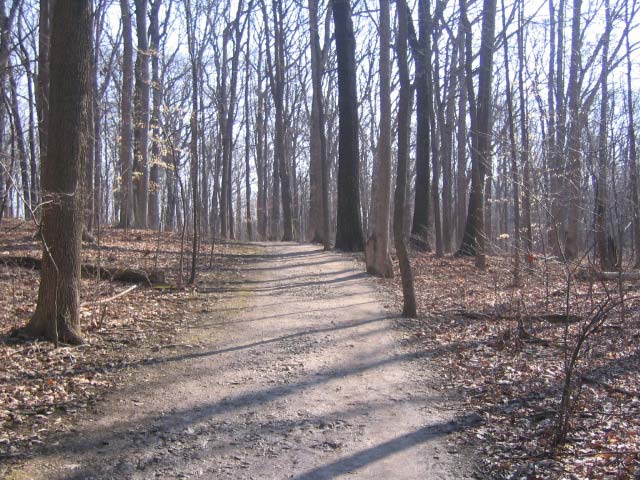

Supplement: Supplementary file 3 [file DataSheet3.ZIP › LowMystery/0_36.jpg]

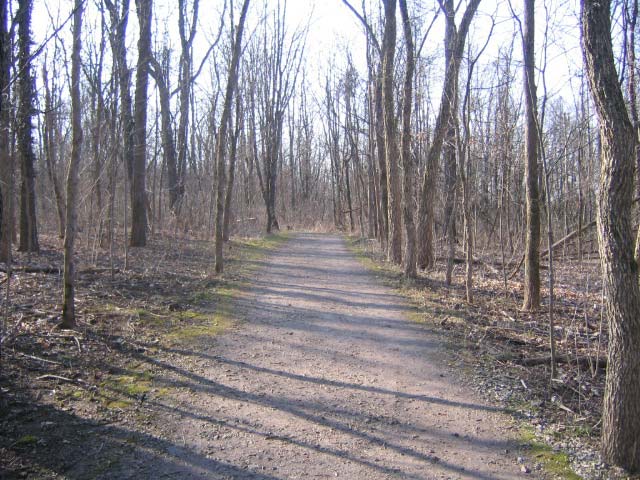

Supplement: Supplementary file 3 [file DataSheet3.ZIP › LowMystery/0_37.jpg]

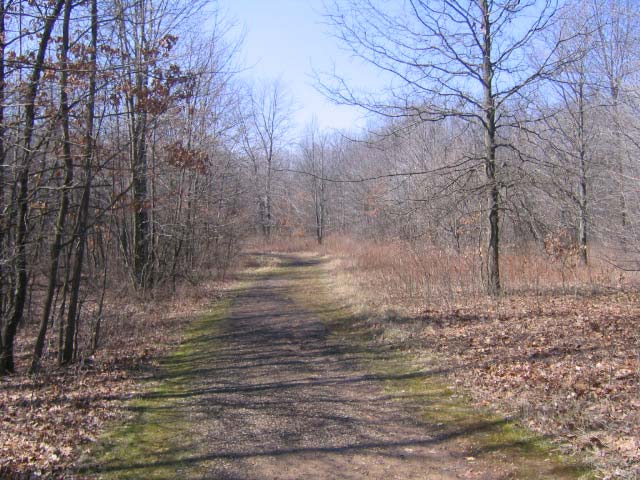

Supplement: Supplementary file 3 [file DataSheet3.ZIP › LowMystery/0_38.jpg]

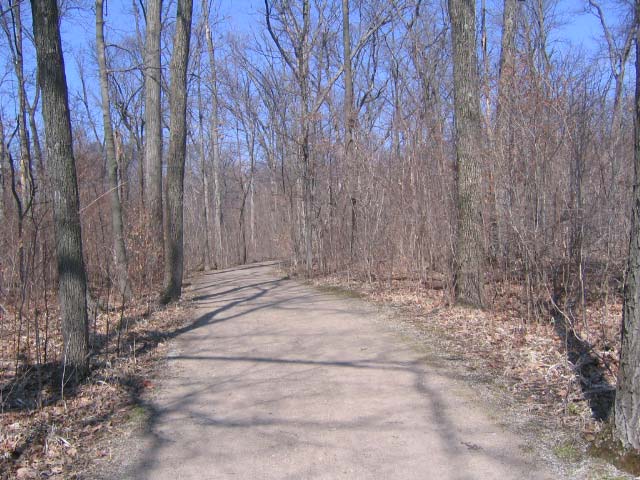

Supplement: Supplementary file 3 [file DataSheet3.ZIP › LowMystery/0_39.jpg]

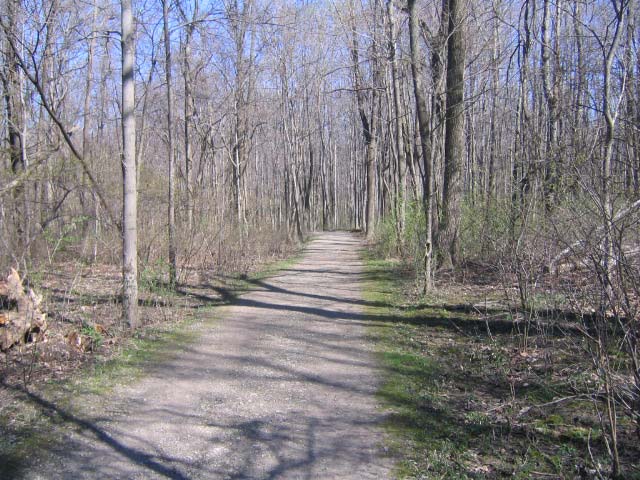

Supplement: Supplementary file 3 [file DataSheet3.ZIP › LowMystery/0_4.jpg]

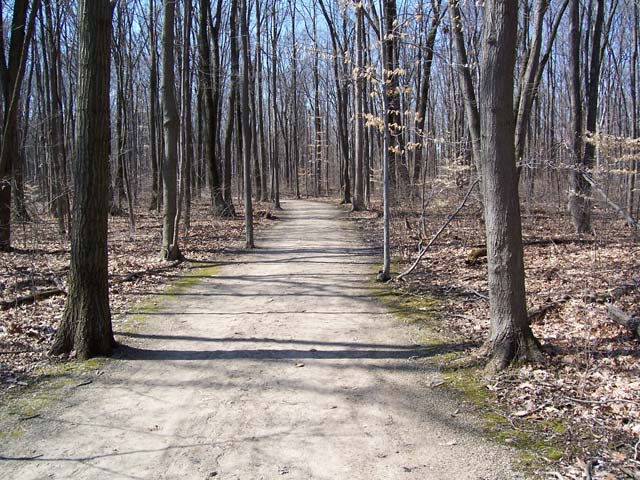

Supplement: Supplementary file 3 [file DataSheet3.ZIP › LowMystery/0_40.jpg]

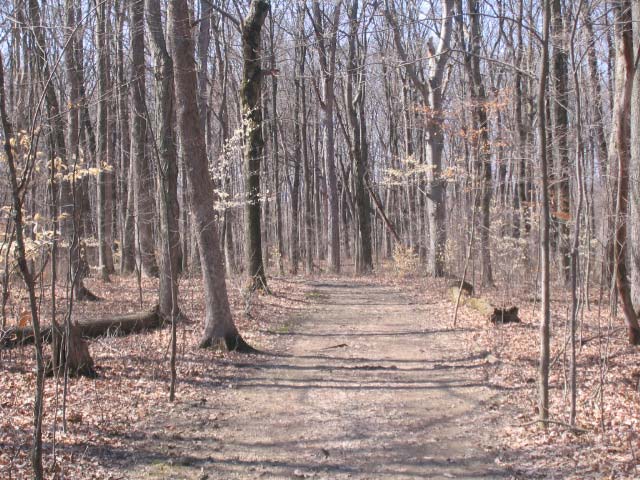

Supplement: Supplementary file 3 [file DataSheet3.ZIP › LowMystery/0_5.jpg]

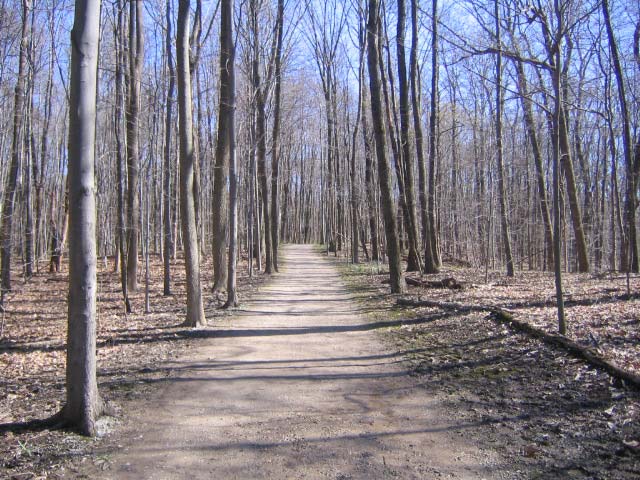

Supplement: Supplementary file 3 [file DataSheet3.ZIP › LowMystery/0_6.jpg]

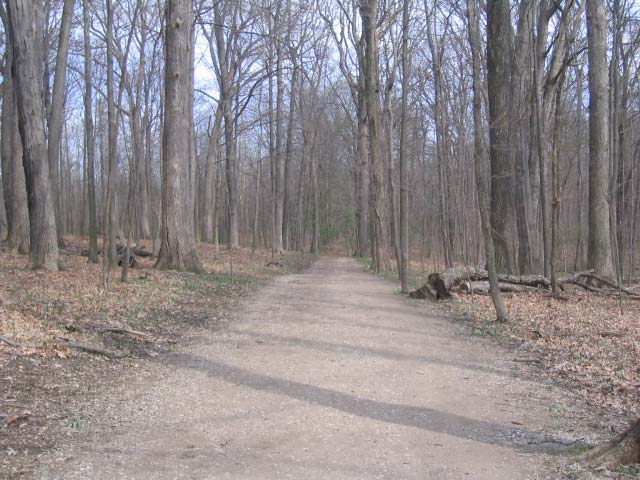

Supplement: Supplementary file 3 [file DataSheet3.ZIP › LowMystery/0_7.jpg]

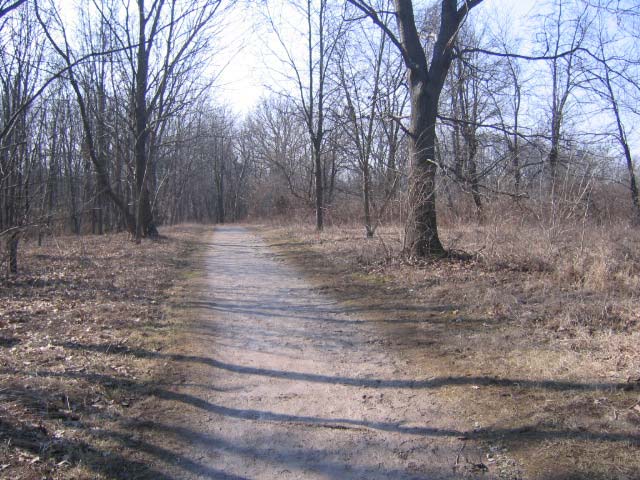

Supplement: Supplementary file 3 [file DataSheet3.ZIP › LowMystery/0_8.jpg]

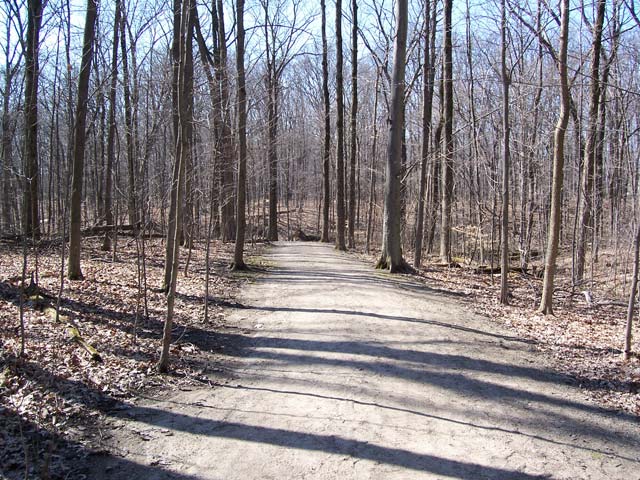

Supplement: Supplementary file 3 [file DataSheet3.ZIP › LowMystery/0_9.jpg]

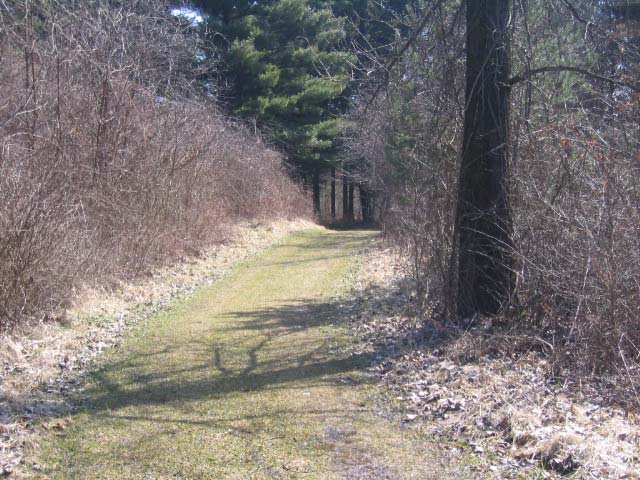

Supplement: Supplementary file 4 [file DataSheet4.ZIP › DataSheet4/HighMystery/1_1.jpg]

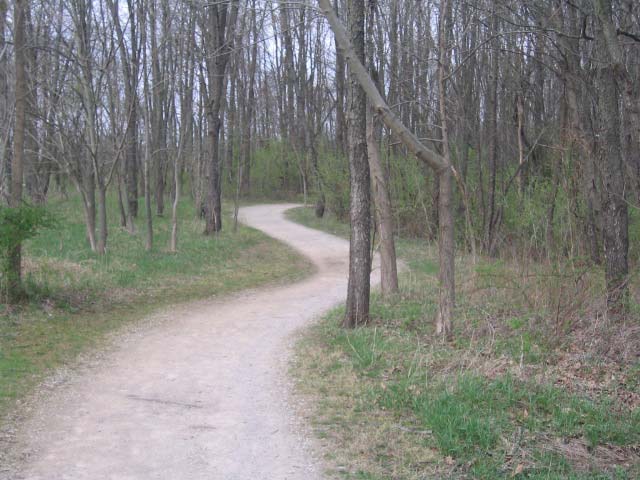

Supplement: Supplementary file 4 [file DataSheet4.ZIP › DataSheet4/HighMystery/1_10.jpg]

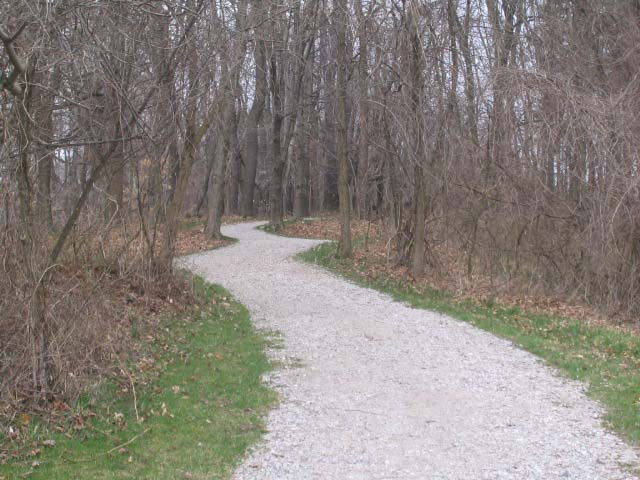

Supplement: Supplementary file 4 [file DataSheet4.ZIP › DataSheet4/HighMystery/1_11.jpg]

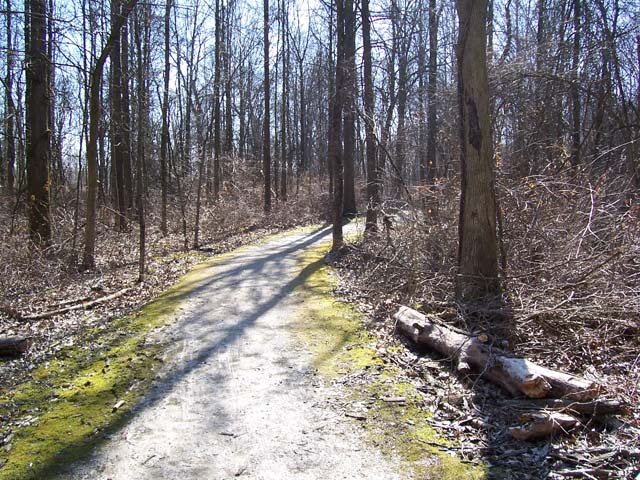

Supplement: Supplementary file 4 [file DataSheet4.ZIP › DataSheet4/HighMystery/1_12.jpg]

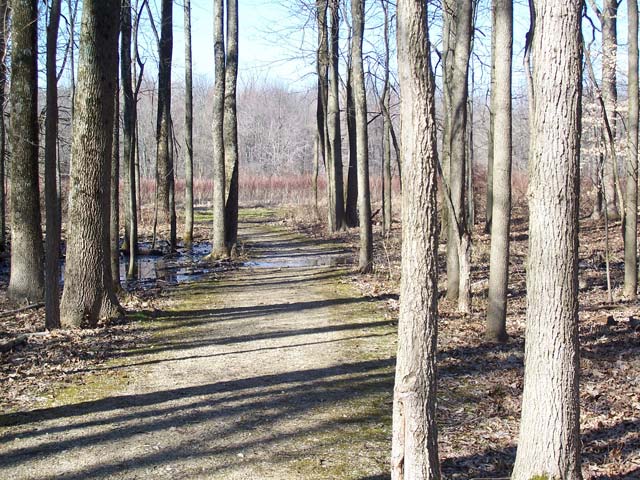

Supplement: Supplementary file 4 [file DataSheet4.ZIP › DataSheet4/HighMystery/1_13.jpg]

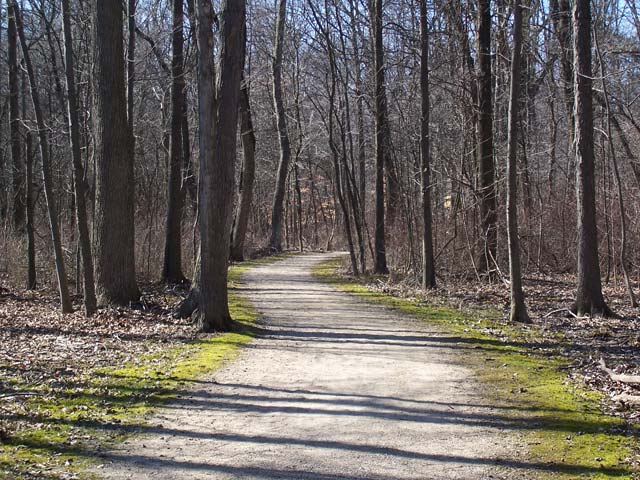

Supplement: Supplementary file 4 [file DataSheet4.ZIP › DataSheet4/HighMystery/1_14.jpg]

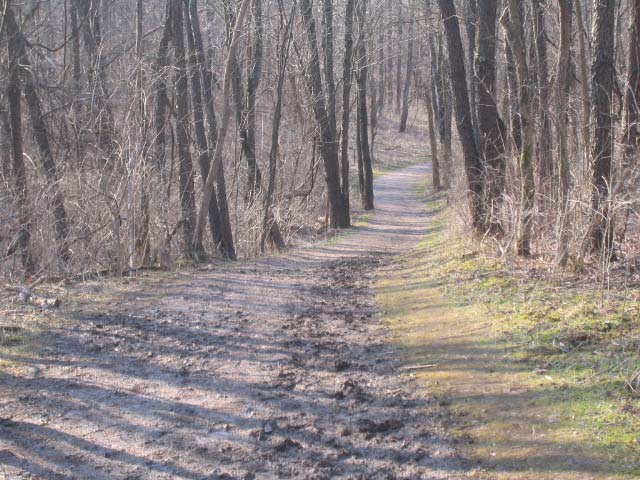

Supplement: Supplementary file 4 [file DataSheet4.ZIP › DataSheet4/HighMystery/1_15.jpg]

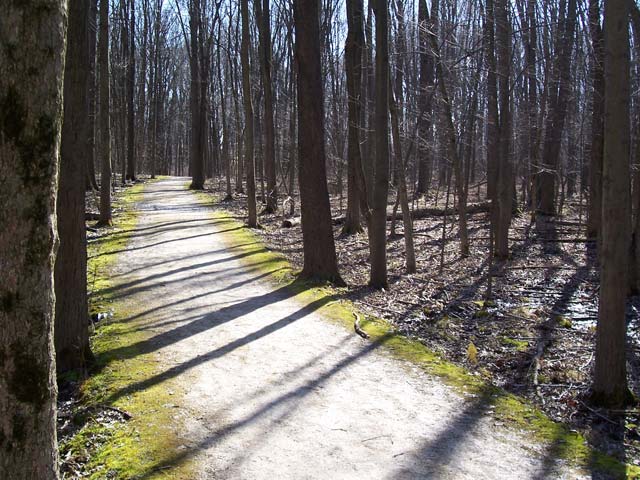

Supplement: Supplementary file 4 [file DataSheet4.ZIP › DataSheet4/HighMystery/1_16.jpg]

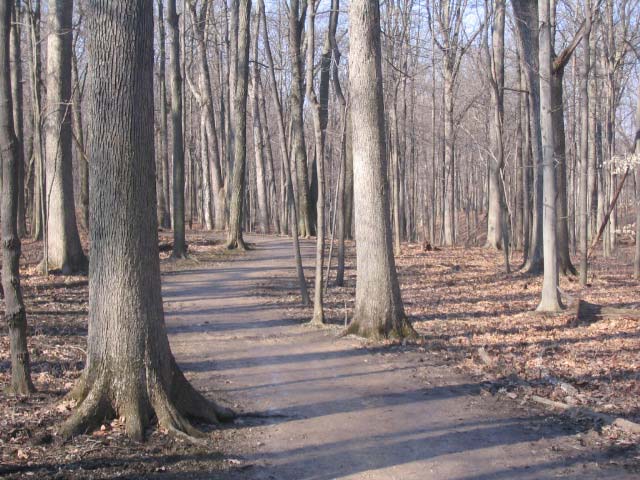

Supplement: Supplementary file 4 [file DataSheet4.ZIP › DataSheet4/HighMystery/1_17.jpg]

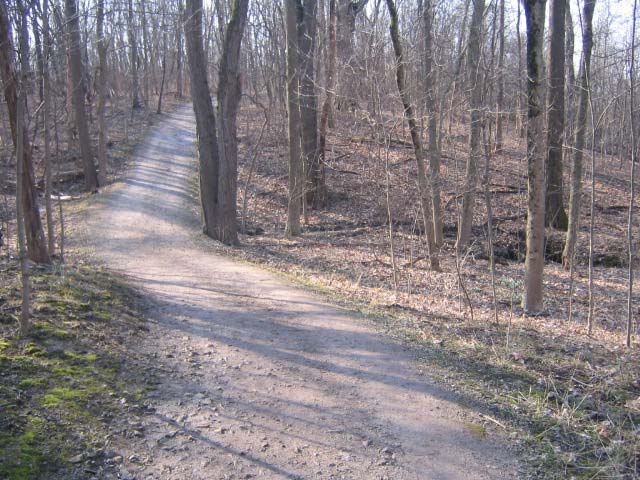

Supplement: Supplementary file 4 [file DataSheet4.ZIP › DataSheet4/HighMystery/1_18.jpg]

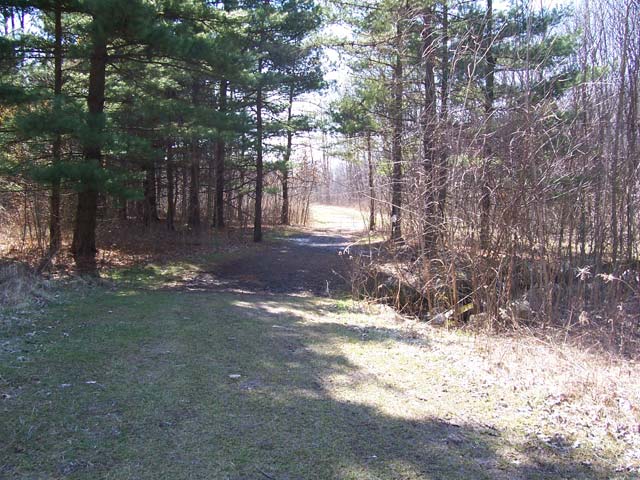

Supplement: Supplementary file 4 [file DataSheet4.ZIP › DataSheet4/HighMystery/1_19.jpg]

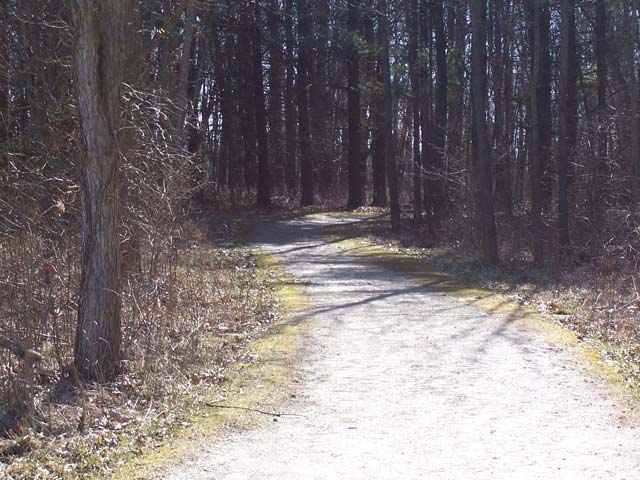

Supplement: Supplementary file 4 [file DataSheet4.ZIP › DataSheet4/HighMystery/1_2.jpg]

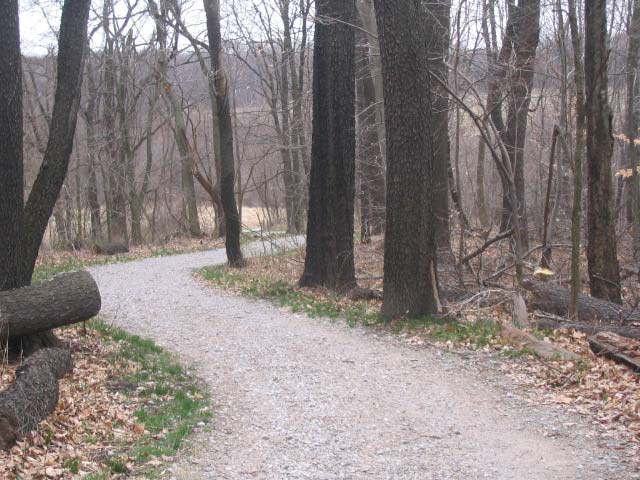

Supplement: Supplementary file 4 [file DataSheet4.ZIP › DataSheet4/HighMystery/1_20.jpg]

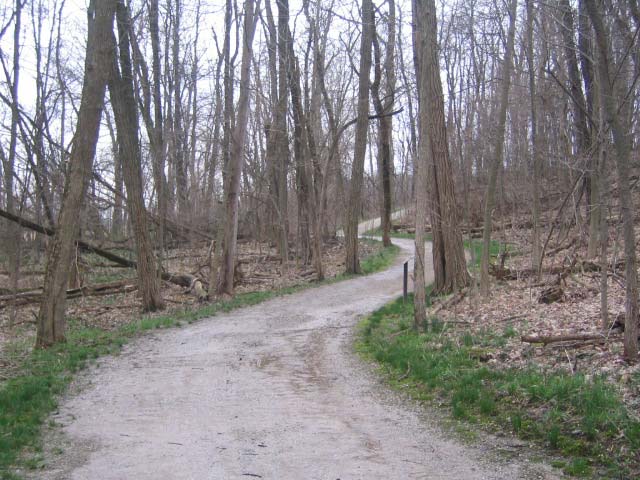

Supplement: Supplementary file 4 [file DataSheet4.ZIP › DataSheet4/HighMystery/1_21.jpg]

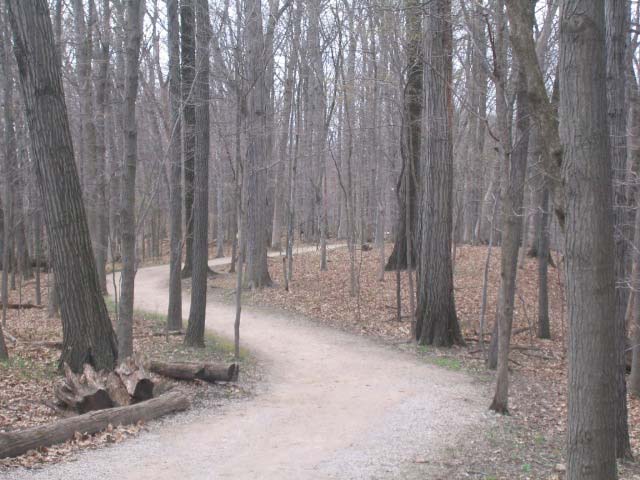

Supplement: Supplementary file 4 [file DataSheet4.ZIP › DataSheet4/HighMystery/1_22.jpg]

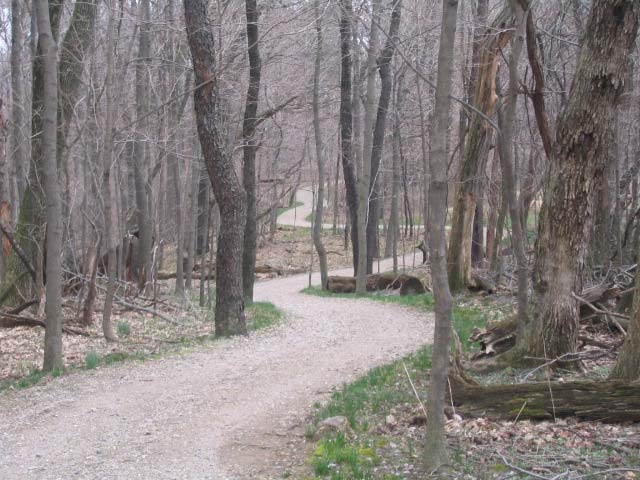

Supplement: Supplementary file 4 [file DataSheet4.ZIP › DataSheet4/HighMystery/1_23.jpg]

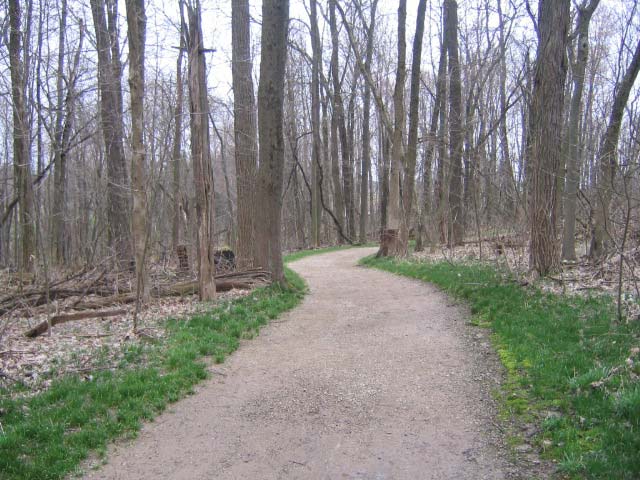

Supplement: Supplementary file 4 [file DataSheet4.ZIP › DataSheet4/HighMystery/1_24.jpg]

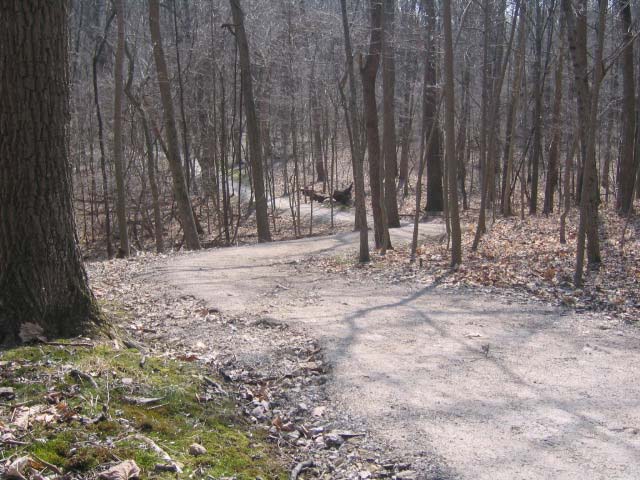

Supplement: Supplementary file 4 [file DataSheet4.ZIP › DataSheet4/HighMystery/1_25.jpg]

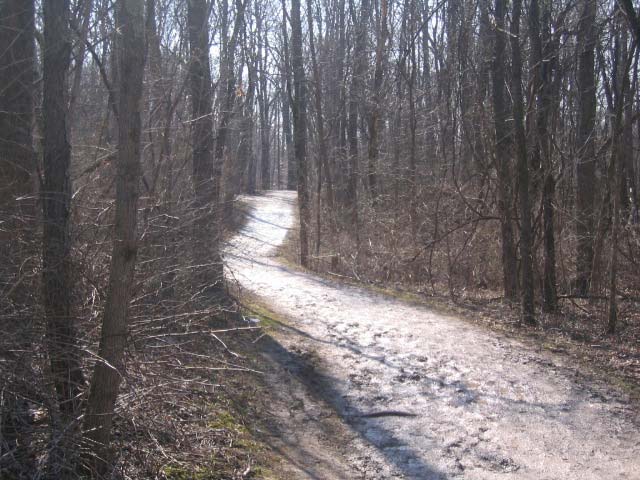

Supplement: Supplementary file 4 [file DataSheet4.ZIP › DataSheet4/HighMystery/1_26.jpg]

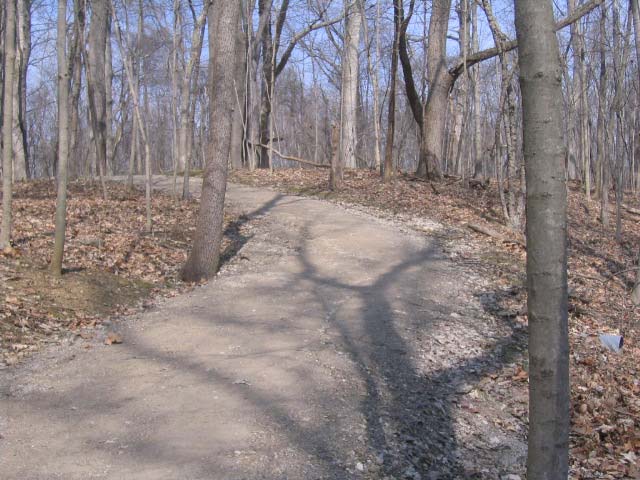

Supplement: Supplementary file 4 [file DataSheet4.ZIP › DataSheet4/HighMystery/1_27.jpg]

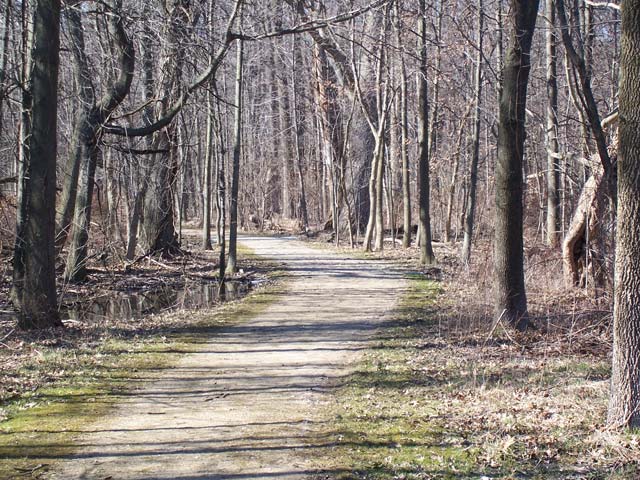

Supplement: Supplementary file 4 [file DataSheet4.ZIP › DataSheet4/HighMystery/1_28.jpg]

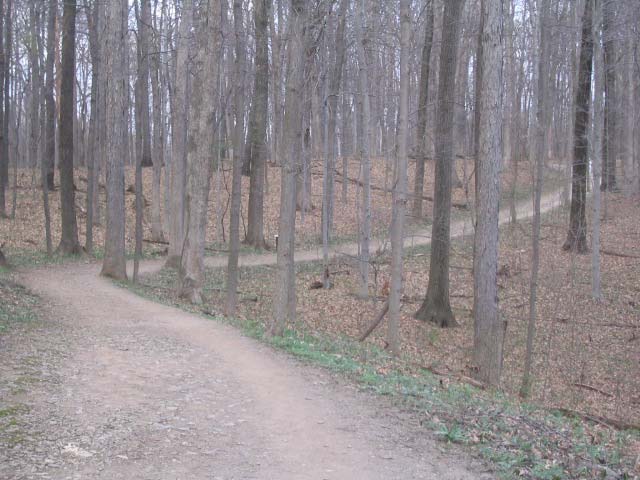

Supplement: Supplementary file 4 [file DataSheet4.ZIP › DataSheet4/HighMystery/1_29.jpg]

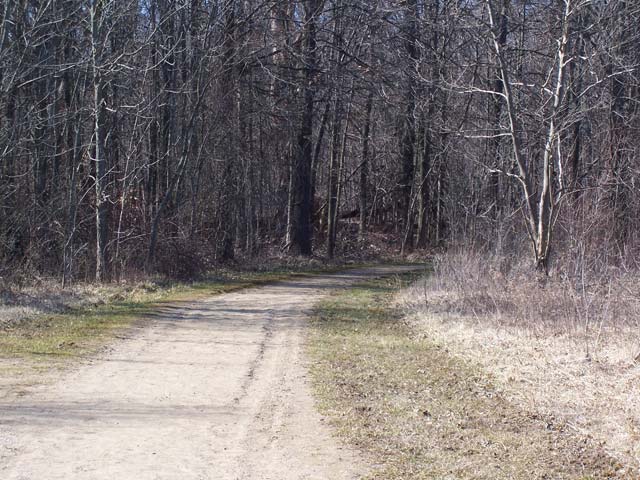

Supplement: Supplementary file 4 [file DataSheet4.ZIP › DataSheet4/HighMystery/1_3.jpg]

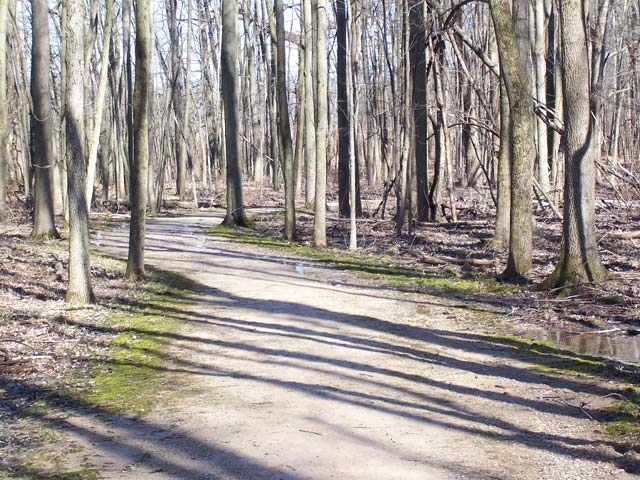

Supplement: Supplementary file 4 [file DataSheet4.ZIP › DataSheet4/HighMystery/1_30.jpg]

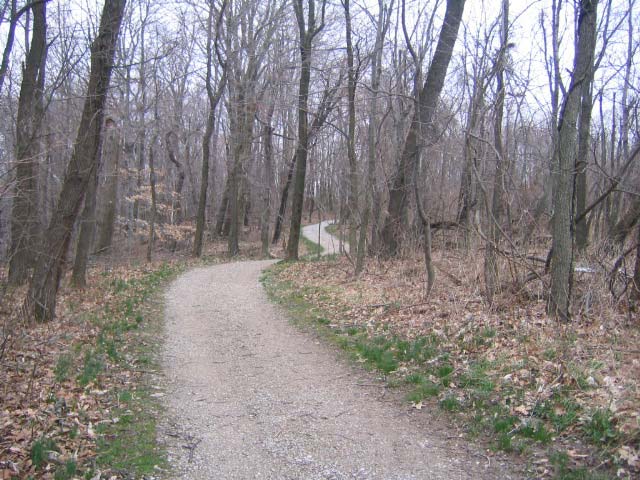

Supplement: Supplementary file 4 [file DataSheet4.ZIP › DataSheet4/HighMystery/1_31.jpg]

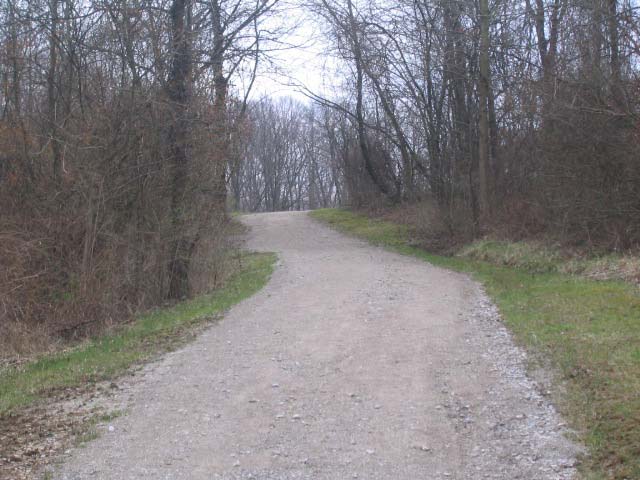

Supplement: Supplementary file 4 [file DataSheet4.ZIP › DataSheet4/HighMystery/1_32.jpg]

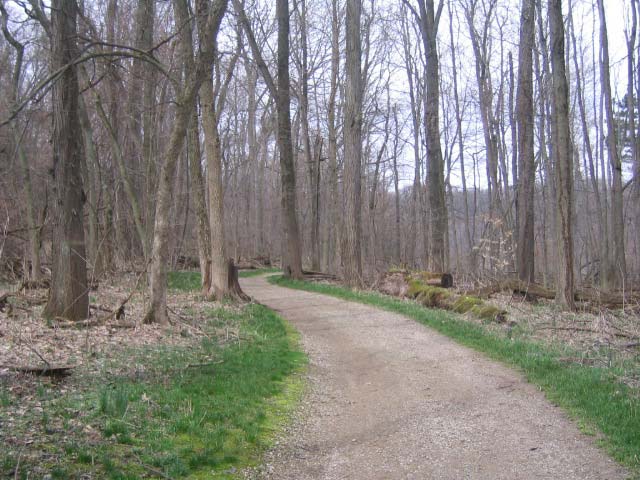

Supplement: Supplementary file 4 [file DataSheet4.ZIP › DataSheet4/HighMystery/1_33.jpg]

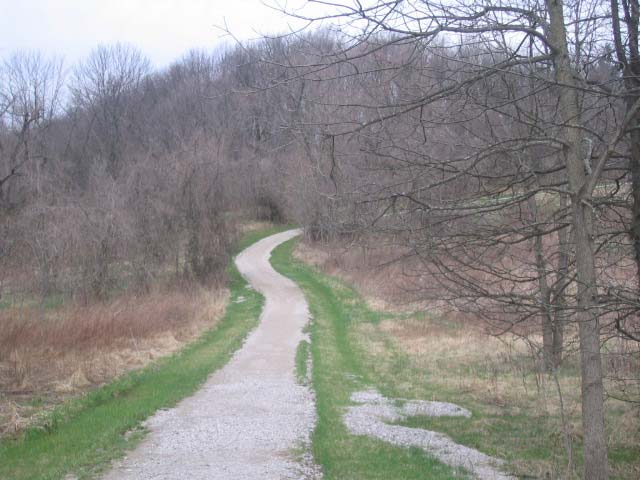

Supplement: Supplementary file 4 [file DataSheet4.ZIP › DataSheet4/HighMystery/1_34.jpg]

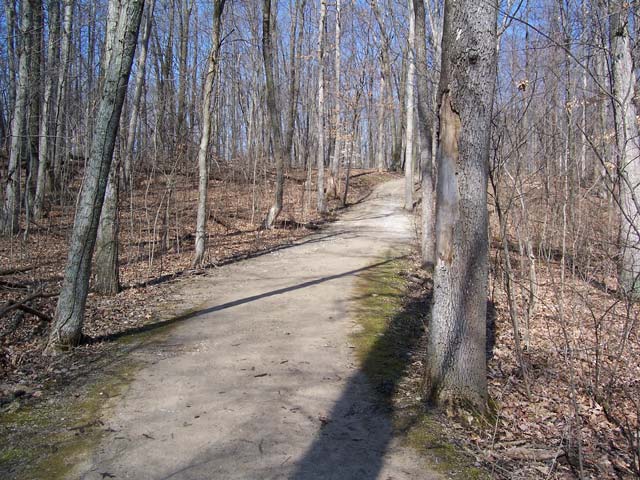

Supplement: Supplementary file 4 [file DataSheet4.ZIP › DataSheet4/HighMystery/1_35.jpg]

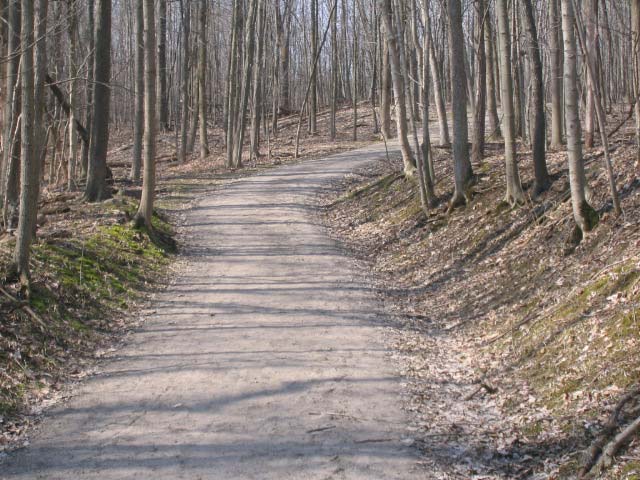

Supplement: Supplementary file 4 [file DataSheet4.ZIP › DataSheet4/HighMystery/1_36.jpg]

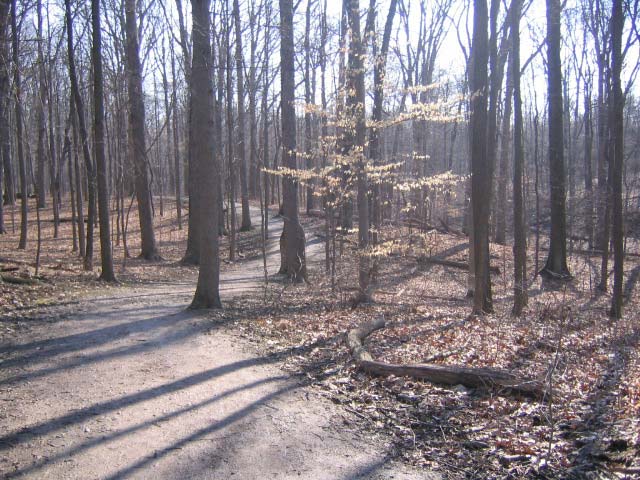

Supplement: Supplementary file 4 [file DataSheet4.ZIP › DataSheet4/HighMystery/1_37.jpg]

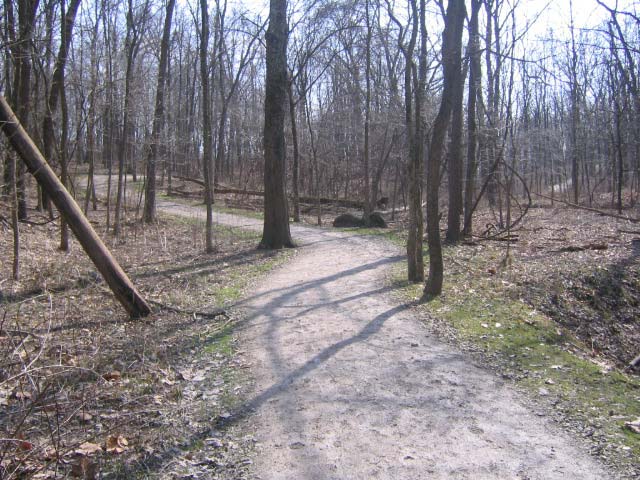

Supplement: Supplementary file 4 [file DataSheet4.ZIP › DataSheet4/HighMystery/1_38.jpg]

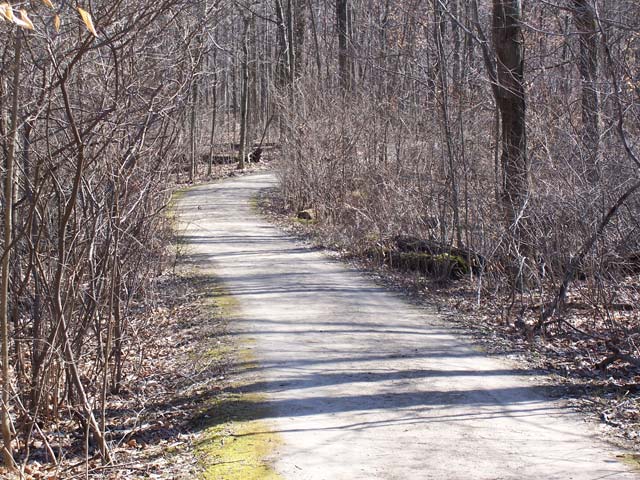

Supplement: Supplementary file 4 [file DataSheet4.ZIP › DataSheet4/HighMystery/1_39.jpg]

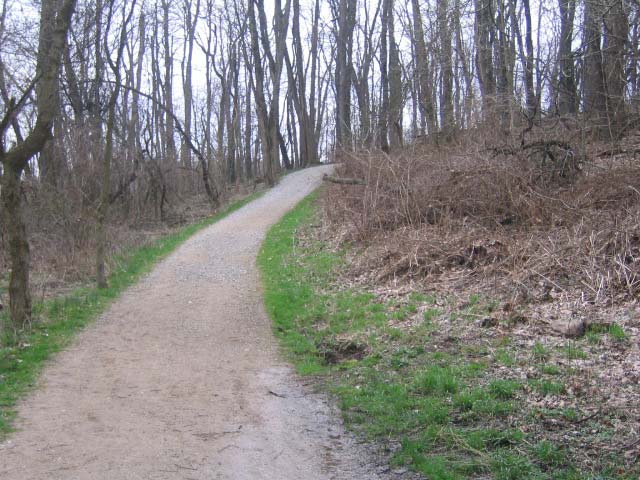

Supplement: Supplementary file 4 [file DataSheet4.ZIP › DataSheet4/HighMystery/1_4.jpg]

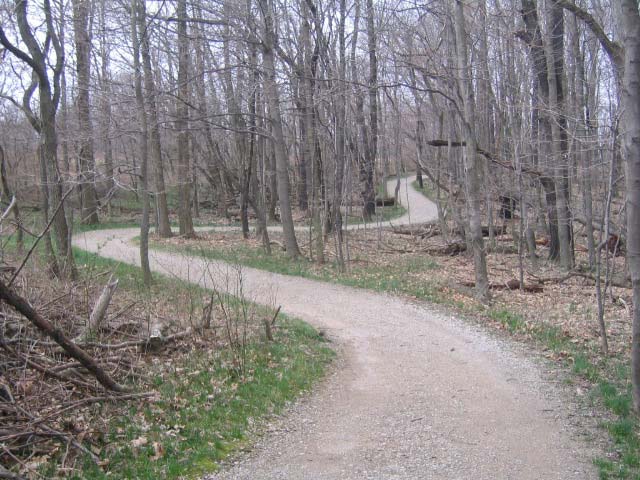

Supplement: Supplementary file 4 [file DataSheet4.ZIP › DataSheet4/HighMystery/1_40.jpg]

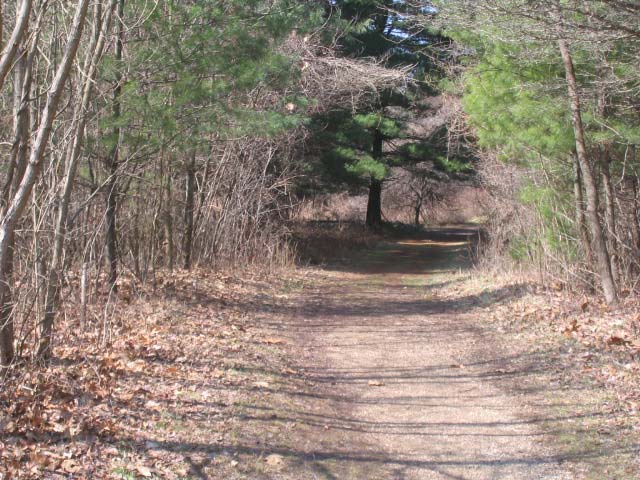

Supplement: Supplementary file 4 [file DataSheet4.ZIP › DataSheet4/HighMystery/1_5.jpg]

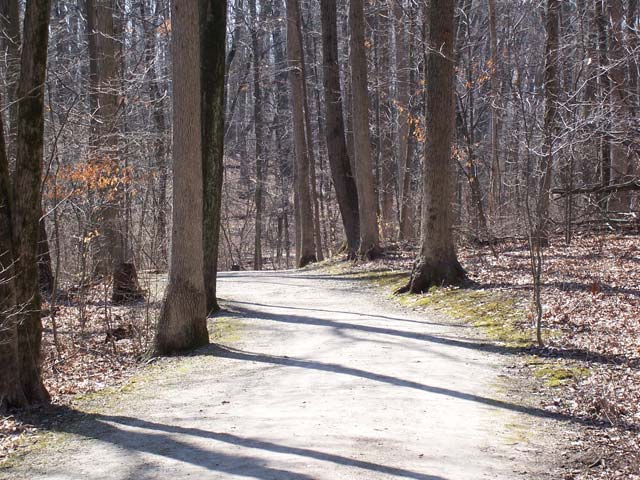

Supplement: Supplementary file 4 [file DataSheet4.ZIP › DataSheet4/HighMystery/1_6.jpg]

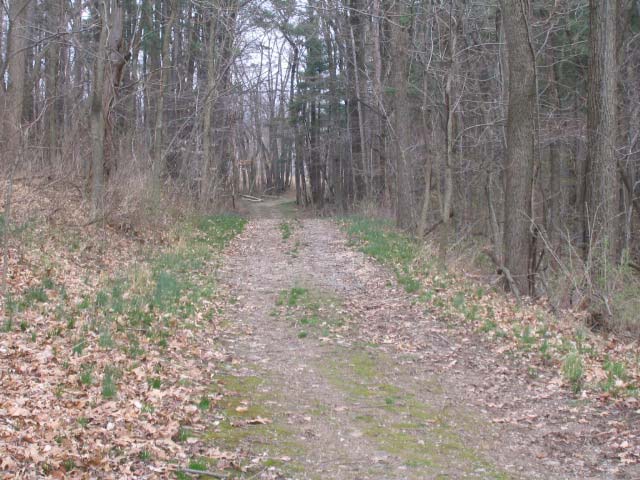

Supplement: Supplementary file 4 [file DataSheet4.ZIP › DataSheet4/HighMystery/1_7.jpg]

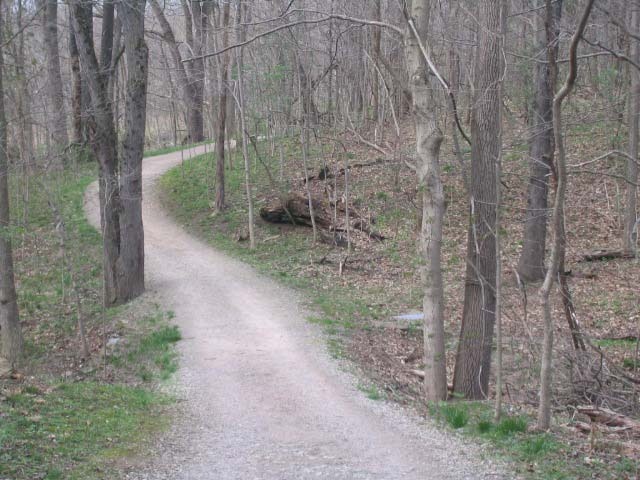

Supplement: Supplementary file 4 [file DataSheet4.ZIP › DataSheet4/HighMystery/1_8.jpg]

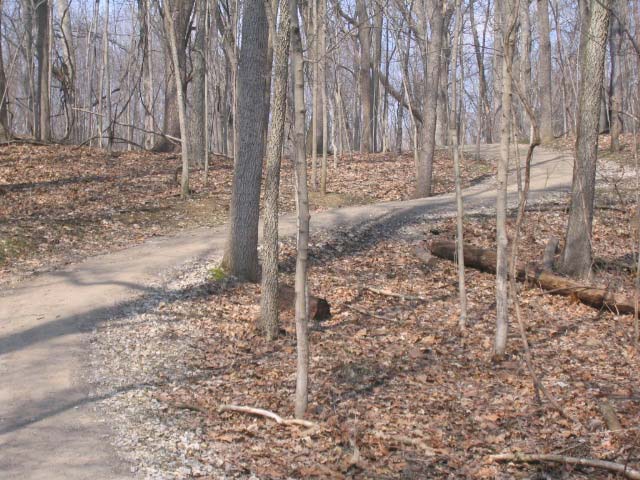

Supplement: Supplementary file 4 [file DataSheet4.ZIP › DataSheet4/HighMystery/1_9.jpg]
